# The elementary step that generates force and sinusoidal analysis in striated muscle fibers

**DOI:** 10.1007/s10974-025-09693-z

**Published:** 2025-07-07

**Authors:** Masataka Kawai

**Affiliations:** Department of Anatomy and Cell Biology, University of Iowa, Iowa City, IA 52242 USA

**Keywords:** Skeletal muscle, Cardiac muscle, Myofibril, Cross-bridge, Endothermic reaction, Lever arm, Power stroke, Cleft closure, Phosphate effect, Kinetics, Rate constant, Transient analysis, Temperature effect, Oscillatory work, Delayed tension, Stretch activation

## Abstract

The elementary step that generates force by cross-bridges (CBs) in striated muscles is reviewed. A literature search focused on models with validating data to verify a CB scheme; models without substantiating data were briefly mentioned or not included. Experimental data include those carried out under the isometric condition in muscle fibers and single myofibrils, along with results from single molecule and stopped-flow studies. These results suggest that force is generated before phosphate (Pi) is released, and the same force is maintained after Pi is released. These studies assumed that Pi is released from myosin. Some results from isotonic experiments are also reviewed, but the data lack the effect of Pi (or a weak effect). Studies with X-ray crystallography and cryo-electron microscopy suggested that force is generated after Pi release from the active site, and Pi is trapped at the secondary site before it is released to the solution. Thus, the difference in the definition of the “Pi release step” must have caused a controversy. It can be concluded that the results from physiological/single molecule studies and cryo-EM/crystal studies complement each other quite well. With isometric experiments, several perturbations are used to generate force transients: length change, chemical change, pressure release, and temperature increase. A small length change includes sinusoidal waveforms, and a large length change includes 10–20% release/restretch. Chemical perturbation includes [Pi] changes. With temperature studies it was shown that the force generation step is endothermic, indicating heat is absorbed. This is qualitatively explained by a hydrophobic interaction between actin and myosin, and by a cleft closure of myosin.

## TABLE OF CONTENTS

**Table:** 

1.	Introduction..................................		
2.	Names of transients..................................		
3.	Experiments using the isometric condition..................................		
	3.1	Experiments performed during steady state..................................	
		3.1.1	Sinusoidal analysis..................................
		3.1.2	Study of phosphate (Pi) effects..................................
		3.1.3	Results from other striated muscle fibers..................................
		3.1.4	CB occupancy at each state..................................
		3.1.5	Identification of CB states..................................
	3.2	Experiments performed during force decrease	
		3.2.1	Caged phosphate experiments in fibers..................................
		3.2.2	Sudden increase in [Pi] in single myofibrils..................................
		3.2.3	Sarcomere collapse(give)..................................
	3.3	Experiments performed during force increase..................................	
		3.3.1	The pressure release experiments..................................
		3.3.2	Unloaded shortening experiment..................................
		3.3.3	*k*_TR_ measurements..................................
		3.3.4	Temperature jump experiments..................................
		3.3.5	Why is the time course slow when force increases?..................................
	3.4	Single molecule studies and stopped flow assays on labelled proteins..................................	
	3.5	Comparison with physiological studies..................................	
	3.6	Pi contamination and the effective Pi concentration..................................	
	3.7	Other evidence of force generation before Pi release..................................	
4.	Experiments performed during isotonic shortening..................................		
	4.1	Force-velocity measurements..................................	
	4.2	Model studies..................................	
5.	Crystallographic and cryo-EM studies..................................		
	5.1	A molecular point of view..................................	
	5.2	Correlation with physiology/single molecule studies..................................	
	5.3	Why is Pi trapped in the secondary binding site?..................................	
6.	Complete CB scheme		
	6.1	Sequence of events..................................	
	6.2	Rate-limiting step and the ATP hydrolysis rate during isometric contraction..................................	
	6.3	Momentum transduction..................................	
	6.4	Force supported by each CB state..................................	
	6.5	Stretch activation, delayed tension, and oscillatory work..................................	
7.	The effect of temperature on the step that generates force..................................		
	7.1	Van’t Hoff equation and enthalpy and entropy changes..................................	
	7.2	Further refinement of Van’t Hoff equation and the heat capacity change..................................	
	7.3	Burial of hydrophobic surface area..................................	
	7.4	Where does unaccounted surface area come from?..................................	
	7.5	Temperature change does not affect force/CB..................................	
8.	Commonalities and differences between physiology and crystal/cryo-EM studies..................................		
9.	Conclusion..................................		
Appendix 1. Analysis of the two-state model (Fig. 1A)..................................			
	A1.1.	Apparent rate constant following to a step change in the experimental condition..................................	
	A1.2.	Response to sinusoidal length changes..................................	
	A1.3.	*k*_TR_ measurement..................................	
Appendix 2. Apparent rate constant of CB scheme in Fig. 1B..................................			
Appendix 3. Simplification of the CB cycle while Step 4 is observed..................................			
Appendix 4. Expanded Van’t Hoff equation..................................			
Appendix 5. Surface area calculation based on *Δ*Cp and *Δ*H°..................................			
References..................................			

## 1. Introduction

Ever since the myosin cross-bridge (CB) was identified as a part of macromolecule that generates force (Huxley and Brown 1967; Huxley 1969), and ATP and myosin were found to cyclically interact with actin to transduce energy (Lymn and Taylor 1971), it has been the focus of our research to determine at which point in the CB cycle force is generated. Solution studies with extracted and reconstituted protein systems have demonstrated that half of ATP hydrolysis energy is used immediately after ATP binding to myosin, and another half is used with phosphate (Pi) release (Taylor 1979; Kodama 1985). Because actin and myosin dissociate after ATP binding, we know that this is not the step that generates force. Consequently, it has been thought that force generation occurs with Pi release. The steps of the CB cycle from solution studies have been reviewed by Taylor (1979), Kodama (1985), Geeves (1991), and Morris and Homsher (1998).

Subsequently, an increasing number of intermediate CB states have been recognized, and their interconversion is called “elementary steps.” Then a question arose: “Does the generation of force occur immediately before or immediately after Pi release?” A line of studies using muscle fibers and myofibrils under the isometric condition have shown that force generation occurs before Pi release. This was determined by identifying the strongly attached AM*ADP.Pi state (Fortune et al. 1991; Kawai and Halvorson 1991; Dantzig et al. 1992; Kawai and Zhao 1993), where A = actin and M = myosin. In contrast, another line of work using crystallographic and cryo-EM studies argued that force generation occurs after Pi release (Llinas et al. 2015; Houdusse and Sweeney 2016); see also Moretto et al. (2022).

I provide an overview of these experiments and their conclusions. I focus on investigations studying muscle fibers and single myofibrils, with which I am much familiar. Some experiments on single molecules are also mentioned, although the protein components and experimental conditions are very different from fiber and myofibril studies. Related subjects were previously reviewed by Takagi et al. (2004) and Stehle and Tesi (2017). In particular, a recent review by Debold (2021) focused on single molecule experiments on power stroke, Robert-Paganin et al. (2020) focused on structural aspects of actomyosin interaction, and Månsson et al. (2023) focused on contraction models. Batters et al. (2014) presented a historical overview focusing on reductionist and comparative approaches, with several key methods developed alongside muscle research. Spudich (2024) also gave a historical review on the development of in vitro motility assays and laser trap experiments on single molecules. As an experimentalist, I will minimize the inclusion of models to only those that are essential for interpreting results; papers that use models without supporting experimental data are not included. Complex models are avoided except in cases where there is a discrepancy between the model and the experimental results.

**Fig. 1:**
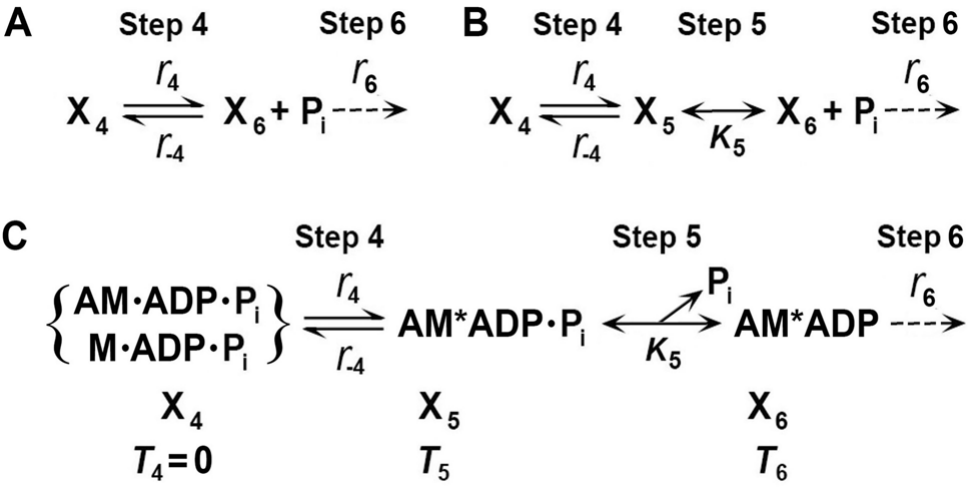
**A** Two-state model (apparent rate constant: Eq. A19), and **B** three-state model (apparent rate constant: Eq. 1) involving phosphate (Pi) release. **C** Three-state model with each molecular constituent identified, where *M* myosin and *A* actin. X_4_, X_5_, and X_6_ represent CB states, and *T*_4_, *T*_5_, and *T*_6_ represent force supported by the respective state. *r*_4_ and *r*_−4_ are the intrinsic rate constants of Step 4, and *K*_5_ is the Pi association constant. Note that *K*_5_ is defined for the X_6_ → X_5_ (backward) direction, hence *K*_5_ is written under the arrows. The slow Step 6 shown with a broken arrow (*r*_6_) appears not to occur while faster Steps 4 and 5 are observed, hence Step 6 can be eliminated from theoretical analysis. Step 6 becomes important when the rate-limiting step is considered

To identify and characterize CB states and the elementary steps between them, it is essential to perturb the CB cycle in such a way that its response is detectable by an observer over a time course. If the observation is focused on tension, the time course is called tension transient. The perturbation can be length change, chemical change, temperature change, etc., but the change must be faster than the transition being observed. The perturbation should be kept to a minimum to avoid complications associated with nonlinear response. These efforts lead to the CB scheme shown in Fig. 1, for the portion surrounding force generation and Pi release steps. In this figure X_4_ is the state that occurs before force generation, and X_6_ is the state that occurs after Pi release. Therefore, X_6_ carries force, whereas X_4_ does not. To start with, we consider four questions. The first question is whether a reported study has identified the intermediate state AM.ADP.Pi, the actomyosin (AM) complex in which ATP is cleaved, while the products ADP and Pi are still located in the myosin head. This intermediate state could exist as two different states, one called the “weakly attached AM.ADP.Pi state” and the other called the “strongly attached AM*ADP.Pi state.” Here, “weakly attached” means without force, and “strongly attached” means with force and indicated by *. In Fig. 1B these states are represented by X_4_ and X_5,_ respectively; X_5_ is absent in Fig. 1A. Solution studies (Taylor 1979; Kodama 1985; Morris and Homsher 1998) lack X_5_; hence, these studies are represented by Fig. 1A and cannot give us the needed answer. The second question is whether the experiment was performed under an isometric condition or an isotonic condition. If an experiment was performed under an isometric condition, then we also want to know whether they were performed while force was developing, declining, or at the steady state. The third question is whether each method has enough resolution to identify elementary steps, and whether the 95% confidence limit was calculated to each fitted parameter to a model. Finally, the fourth question is if a large diameter of fiber preparation leads to misleading information, as implied by Debold (2021).

## 2. Names of transients

Because different authors have used different nomenclatures and terminologies for different types of perturbations and transients while investigating CB mechanisms of contraction, it may be proper to first summarize their correlations in Table 1. Transients with a medium speed are Pi sensitive and likely reflect the force generation step, so they are listed in the middle column. Slower transients are placed in the left column, and faster transients are placed in the right column.

**Table 1:** Correlation of the force transients used by various authors, except for *length transient

Authors	Perturbations	Slow	Medium	Fast	Instantaneous
Kawai and Halvorson (1991)	Length, sinusoidal	Process A	Process B	Process C	*Y*_∞_
Fortune et al. (1991)	Pressure release	Phase 3	Phase 2		Phase 1
Dantzig et al. (1992)	Caged Pi	Phases III + IV	Phase II	Phase I	
Walker et al. (1992)	Caged Pi	Slow phase	Fast phase (*k*_Pi_)		
Wahr et al. (1997)	Large release/restretch		*k*_tr_		
Ranatunga (1999a)	Temperature jump	Phase 2	Phase 1		
Caremani et al. (2008)	Length release, 3%	Slow exp (*r*_s_)	Fast exp (*r*_f_)		
Caremani et al. (2013)*	Force decrease	Phase 4	Phase 3	Phase 2	Phase 1

Pi-sensitive transients are listed in the middle column, labelled “Medium.” Slower transients are listed in the left column, and faster transients are listed in the right column

*Exp* exponential

## 3. Experiments using the isometric condition

To begin with, it may be necessary to define what “isometric” means. It literally means that both ends of the muscle preparation are fixed, so that muscle length does not change while an experiment is performed. This situation is in contrast to “isotonic” experiments, in which the muscle continuously shortens under a constant load. Consequently, for most applications isometric means that the length is fixed and does not change over time. Sometimes a segmental length is fixed (changing the overall length), which is a desirable measurement when the contraction is heterogeneous; in this case the force is the same throughout the length of the preparation. This requires technical skill. In intact right ventricular trabecular fibers with twitch contraction, Caremani et al. (2016) observed a local sarcomere shortening, which they compensated by stretching for the next twitch, resulting in a larger force development as expected. In single myofibril experiments from rabbit psoas Iorga et al. (2012) closely examined the sarcomere length during Ca activation, but a similar local shortening did not occur.

One advantage of isometric experiments is that CBs are arranged in parallel in the half sarcomere, where all of the elementary steps of the CB cycle take place. Under this condition, there is a possibility of resolving individual elementary steps because CB force is additive in the half sarcomere. This can be seen in 3–4 exponential processes in skeletal muscle fibers (Huxley 1974; Kawai and Brandt 1980; Kawai and Zhao 1993; Wang and Kawai 1997; Galler et al. 2005; Kawai et al. 2018) or 2–3 exponential processes in cardiac muscle strips (Kawai et al. 1993; Wannenburg et al. 2000; Lu et al. 2006). Each one of these exponential processes corresponds to an elementary step in the CB cycle. Because our interest is to resolve the elementary steps of the CB cycle, we chose to use the isometric condition.

Since results may not necessarily be the same if measurements were carried out during a force increase, a force decrease, or a steady state, such as experienced by Tesi et al. (2000), they are discussed separately in the following.

### 3.1. Experiments performed during steady state

I will first review results obtained from sinusoidal analysis, because I am much involved in this analysis method in my research, and I believe that this is one of the best methods to characterize the elementary steps of the CB cycle in structured muscle fiber and myofibril systems. When this analysis method is computerized, it is also an efficient method to produce a large amount of data inexpensively in a short time. I will then compare these results with experiments performed by other investigators. Our strategy is first to measure the apparent rate constants as functions of [Pi] and then to establish the CB scheme together with kinetic constants (intrinsic rate and equilibrium constants). Second, we use these kinetic constants to calculate the occupancy (probability) of CBs at each state as a function of [Pi]. Third, we then correlate the occupancy with measured force as a function of [Pi] to deduce force supported by each CB state.

#### 3.1.1. Sinusoidal analysis

The length change applied during the sinusoidal analysis is very small, in the range of 0.125–0.2% of muscle length, which translates to ≤ 1.25 nm at the CB level when 50% series compliance is considered (Huxley et al. 1994; Wakabayashi et al. 1994). If the length change exceeds the CB’s step size (5.3–12.6 nm) (Kitamura et al. 1999; Lombardi et al. 2004; Sherwood et al. 2004; Wu and Nakamura 2005), then the CB cycles more than once, and the data collected become influenced by the slowest step (~ 16 s^−1^ in rabbit psoas at 20°C) of the cycle, making it more difficult to resolve faster elementary steps.

Sinusoidal analysis is carried out when force is at a plateau. In many muscles, sinusoidal force change becomes steady (stable and reproducible) after 0.25 s of oscillation, at which point the force and length time courses are collected simultaneously at the maximum speed (every 10 μs with two A/D converters). The 0.25-s duration is chosen because a solution to a linear differential equation with forced oscillation has two components: one transient and the other steady sinusoidal oscillation (see Appendix 1, Eq. A18). The transient is caused by a sudden application of the sinusoidal length change—a similar situation to the step-length-change experiments used by Huxley and Simmons (1971). I assumed that the transient is over in 0.25 s. The signal is averaged for each sine cycle during which time the data are collected, and the time course is analyzed by Fourier transform to deduce elastic and viscous moduli (or amplitude and phase shift) (Kawai and Brandt 1980). Clearly, the more data points that are collected, the better the results. This method is free of the problems associated with force rise or decay, described later (Sections 3.2.3 and 3.3.5). At the same time, it is a steady state measurement, hence the signal-to-noise ratio (S/N) can be improved by prolonging the data collection period. That is, sinusoidal analysis inherently incorporates a signal averaging procedure. As a result, the data are generally more accurate compared to transient analysis on force time courses (these are compared in Kawai 1986); both of these methods seek the same information—“apparent rate constants”—in exponential processes, to establish a sequence of events in the CB cycle.

#### 3.1.2. Study of phosphate (Pi) effects

**Fig. 2:**
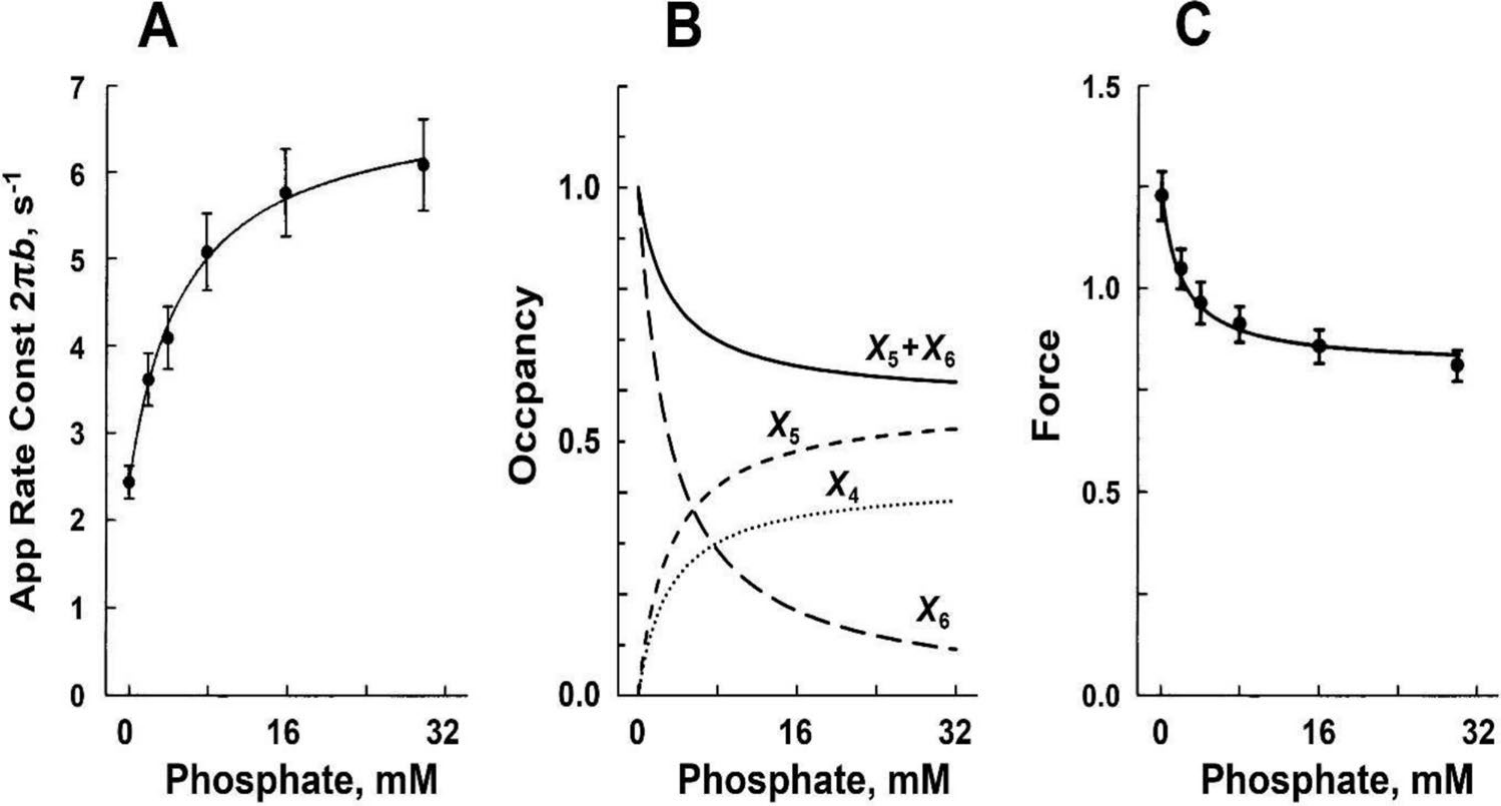
Experiments on rabbit soleus slow-twitch fibers, activated at pCa 4.40 and 5 mM MgATP at 20°C. The average over nine experiments, with SEM, are shown. **A** Apparent rate constant 2π*b* is plotted against [Pi] and fitted to Eq. 1. From this fitting, three kinetic constants (*r*_4_ = 5.7 ± 0.5 s^−1^, *r*_−4_ = 4.5 ± 0.5 s^−1^, and *K*_5_ = 0.18 ± 0.01 mM.^−1^) were deduced. **B** Changes in CB occupancy *X*_4_ (Eq. 2), *X*_5_ (Eq. 3), and *X*_6_ (Eq. 4) as functions of [Pi], calculated by using *K*_4_ (≡*r*_4_/*r*_−4_ = 1.37 ± 0.13) and *K*_5_ obtained from **A**. Also plotted is *X*_5_ + *X*_6_ (labelled). **C** Isometric force (normalized to first control force *T*_c_) is plotted against [Pi] and fitted to Eq. 6 by using *K*_4_ and *K*_5_ deduced from **A**. From this fitting, *T*_5_ = *T*_6_ = 1.29 ± 0.05 *T*_c_ (N = 9) was obtained, where *T*_c_ = 142 ± 15 kPa (N = 24). In **A** and **C**, the curved lines are the best fit results; reproduced from Wang and Kawai (1997)

In sinusoidal analysis with frequencies ranging from 0.25 to 350 Hz (0.5–650 ms in time domain) in skeletal muscle fibers, three exponential processes A, B, and C were identified, with the apparent (= observed) rate constants 2π*a*, 2π*b*, and 2π*c*, respectively (Kawai et al. 1977; Kawai and Brandt 1980). Of these, 2π*b* was studied as the function of [Pi] (Fig. 2A). Compared to 2π*a* and 2π*c*, 2π*b* changes most significantly with [Pi] (Kawai 1986). These experiments have demonstrated that 2π*b* increases with [Pi] and approaches a saturation toward 30 mM [Pi] as shown in Fig. 2A (Kawai and Halvorson 1991; Wang and Kawai 1997). From the plot in Fig. 2A, a three-state model was constructed (Fig. 1B), and two intrinsic rate constants (*r*_4_, *r*_−4_), and the Pi binding (association) constant (*K*_5_) were deduced by fitting the apparent rate constant data 2π*b* to Eq. 1:

1 $$2\uppi b={r}_{4}+\frac{{K}_{5}P}{1+{K}_{5}P}{r}_{-4}.$$

Equation 1 is the analytical form (derived in Appendix 2, Eq. A28) of the apparent rate constant (2π*b*) measured in the CB scheme shown in Fig. 1B (Kawai and Halvorson 1991), where *P* = [Pi]. Equation 1 relates intrinsic rate constants (*r*_4_, *r*_−4_) and the Pi association constant (*K*_5_) to the apparent rate constant (2π*b*). Here we assume that in fibers and/or myofibrils, elementary Steps 4 and 5 are reversible as shown in solution studies (Bagshaw and Trentham 1973), in fiber studies (Hibberd et al. 1985; Webb et al. 1986), and in cryo-EM studies (Llinas et al. 2015). We also assume that Pi binding and/or release (Step 5) is faster than Step 4; hence, Step 5 can be approximated by an equilibrium while Step 4 is measured at frequencies around *b*.

By fitting the result of *P* vs. the apparent rate constant 2π*b* to Eq. 1, two intrinsic rate constants (*r*_4_, *r*_−4_) and the Pi association constant (*K*_5_) of the elementary steps are deduced. The data fit well, as shown by the curved line in Fig. 2A. Once *K*_5_ is obtained (found by minimizing the sum of squares by iteration), Eq. 1 is linearly related to *r*_4_ and *r*_−4_; hence, these are readily deduced by the standard linear fitting procedure, together with the 95% confidence limits on fitted parameters. From this fitting we obtained the following for rabbit psoas fibers: *r*_4_ = 56 ± 2 s^−1^; *r*_−4_ = 129 ± 10 s^−1^; *K*_4_ = 0.45 ± 0.05; and *K*_5_ = 0.069 ± 0.007 mM^−1^ (Table 2) (Kawai and Halvorson 1991), and for rabbit soleus slow-twitch fibers (Fig. 2A): *r*_4_ = 5.7 ± 0.5 s^−1^; *r*_−4_ = 4.5 ± 0.5 s^−1^; *K*_4_ = 1.37 ± 0.13; and *K*_5_ = 0.18 ± 0.01 mM (Table 3) (Wang and Kawai 1997), where numbers after ± represent SEM; the maximum error is 11%. An important reminder here is that only three parameters were extracted from the *P* vs. rate constant results; therefore, the 95% confidence limits of fitted parameters are narrow (usually less than ± 15% of the value), hence the reliability of these parameters is very high. This is because Fig. 2A is based on the average of nine experiments (9 × 6 = 54 total data points); therefore, the degree of freedom is *N*_DF_ = 54 − 3 − 1 = 50 (three fitting parameters and one average point are subtracted) and is large. The equilibrium constant *K*_4_ of Step 4 is calculated from *r*_4_ and *r*_−4_ (*K*_4_≡*r*_4_/*r*_−4_).

**Table 2:** Results of kinetic studies on the Pi release step in rabbit psoas fibers and ^†^psoas myofibrils, studied under the isometric condition

	*r*_4_	Adj *r*_4_	*r*_−4_	*K*_4_≡ *r*_4_/*r*_−4_	1/*K*_5_ mM	±	Temperature	Methods	Transient name
Publications	s^−1^	s^−1^	s^−1^				°C		
Kawai and Halvorson (1991)^#^	56	56	129	0.45	14.5	x	20	Sinusoidal *ΔL*	Process B
Fortune et al. (1991)	16.88	62	~ 33	~ 0.51	3.92	–	12	Pressure release	Phase 2
Dantzig et al. (1992)	79.2	79.2	114.7	0.690	3.7	‒	20	Caged Pi	Phase II
Walker et al. (1992)	27	61	115	0.23	6.1	‒	15	Caged Pi	*k*_Pi_
Zhao and Kawai (1993)	106	106	90	1.20	5.3	x	20	Sinusoidal *ΔL*	Process B
Wahr et al. (1997)	12	27	44	0.27	7	‒	15	Release/restretch	*k*_tr_
Ranatunga (1999a)	49.5	181	135	0.367	8.0	x	12	T-jump, 9 → 12 °C	
Tesi et al. (2002a)^†^	20	45	250	0.08	64	‒	15	Solution switch	*k*_Pi(+)_
Caremani et al. (2008)	21.2	77	66.4	0.319	28.9	x	12	3% Length release	*r*_F_
Kawai and Iorga (2024)^†^	104	234	282	0.37	8.3	x	15	Sinusoidal *ΔL*	Process B

For definitions of rate constants (*r*_4_, *r*_−4_) and equilibrium constants (*K*_4_, *K*_5_), refer to Fig. 1B/C. “ ~ ” indicates that the data were estimated from published figures. Other data were calculated based on published values. Order is based on the publication year/month. Q_10_ of *r*_4_ is reported to be 3.75 (Dantzig 1992) and 6.8 (Zhao and Kawai 1994); their geometric average is: Q_10_ = 5.05. The column labelled Adj *r*_4_ includes *r*_4_ values that were adjusted to those at 20 °C by using the averaged Q_10_ value. All of these publications agree to the CB scheme in Fig. 1B/C, which indicate that force is generated in Step 4 before Pi is released, and the same force is maintained after Pi release

± Statistics (*x* performed, –not performed), *ΔL* length change

^#^Submitted February 12, 1990

Table 2 summarizes results obtained on rabbit psoas fibers/fibrils from several different laboratories with many different techniques. Their experimental details are discussed in Sects. 3.2–3.3. For ease of comparison, the value of *r*_4_ is adjusted to that at 20°C by using Q_10_, and listed it in a column labelled Adj *r*_4_ in Table 2. This is because different experiments were carried out at different temperatures, which have a large effect on the rate constants. Q_10_ of *r*_4_ is reported to be 3.75 (Dantzig et al. 1992) and 6.8 (Zhao and Kawai 1994). Their geometric average is Q_10_ = 5.05, which results in Q_8_≡Q_10_^0.8^ = 3.65 and Q_5_≡Q_10_^0.5^ = 2.25. This results in *r*_4_ to be in the range of 45–106 s^−1^ for most reports in Table 2. An exception is 27 s^−1^ (Wahr et al. 1997), because *k*_TR_ was used, which gives a low value, as described in Sect. 3.3.5. Another exception is 181 s^−1^ (Ranatunga 1999a) with T-jump experiments, which may be affected by faster steps in the CB cycle. Our single myofibril experiment gave 234 s^−1^ (Kawai and Iorga 2024), which was also high because force was used for the calculation of *r*_4_, and the equilibrium in Fig. 6 was considered to be *K*_3c_ = 0.1, resulting in a high value of *r*_4_.

#### 3.1.3. Results from other striated muscle fibers

The sinusoidal analysis method was applied to other striated muscle fibers: three types of fast-twitch fibers; slow-twitch fibers; and cardiac fibers; the results are summarized in Table 3. The results of sinusoidal analysis (frequency plots of the complex modulus, such as Nyquist plots) correlate perfectly well with biochemical analyses of fiber types by using myosin heavy chains (Galler et al. 2005) and light chains (Kawai and Schachat 1984); their characteristics were summarized in Figs. 4-4 and 4-5 of Kawai (2018b). Consequently, the fiber type can be determined as soon as sinusoidal analysis is finished (frequency plots are displayed on the computer screen 1 s after the measurement), without carrying out gel electrophoresis. This quick determination of the fiber type facilitates experiments, because an investigator can decide on next experiments while the fiber is still active in the experimental chamber. Correlation between fiber types and myosin heavy chain isoforms was experimentally shown in a solution study (Weiss et al. 2001).

**Table 3:** Results of kinetic studies on the Pi release step in various fiber types, mostly based on the rate constant 2π*b* of Process B

	*r*_4_	*r*_−4_	*K*_4_≡*r*_4_/*r*_−4_	*K*_5_	±	Temperature	Muscle samples
Publications	s^−1^	s^−1^		mM^−1^		°C	
*Fast-twitch skeletal fibers*							*Fibers, myofibrils*^*†*^
Galler et al. (2005)	143	81	2.03	0.26	x	20	Rabbit AMM (IIB)
	58	63	1.11	0.16	x	20	Rabbit AMM (IID)
	13.6	13.6	1.21	0.18	x	20	Rabbit EDL (IIA)
*Slow-twitch skeletal fibers*							
Kawai et al. (2018)	6.9	34	0.52	0.14	x	25	Mouse soleus
Wang and Kawai (1997)	5.7	4.5	1.37	0.18	x	20	Rabbit soleus
Tesi et al. (2002a)	3	3.3	0.92	0.1	–	15	Rabbit soleus^†^
Millar and Homsher (1992)	1.96				‒	20	Rabbit soleus
*Myocardium*							*Fibers, myocytes*^*‡*^
Wang et al. (2013)	113	372	0.34	0.066	x	20	Mouse papillary
Kawai et al. (2017)	45	117	0.56	0.19	x	25	Mouse papillary
Xi et al. (2022)	49	77	1.07	0.172	x	25	Mouse papillary
Kawai et al. (1993)	11	107	0.11	0.060	x	20	Ferret trabecular
Kawai et al. (2016)	15.0	142	0.118	0.148	x	25	Rat papillary α
	9.8	31.6	0.393	0.145	x	25	Rat papillary β
Araujo and Walker (1996)	5	45	0.11	0.067	‒	15	Rat ventricle‡
Zhao and Kawai (1996)	3.2*	10.5	0.32	0.104	x	20	Porcine myocardium
Fujita et al. (2002)	7.1	13	0.59	0.14	x	25	Bovine myocardium
Bai et al. (2011)	5.9	7.4	0.96	0.58	x	25	Bovine myocardium

Order is based on animal size and then on the rate constant *r*_4_

*AMM* adductor magnus muscle, *EDL* extensor digitorum longus muscle. ± Statistics (*x* performed, –not performed)

Single myofibrils (^†^) or myocytes (^‡^) were used for experiments

*This becomes 7.2 s^−1^ at 25 °C with Q_5_ = 2.25

Among cardiac muscle fibers, it is noticeable that the rate constants are faster in smaller mammals than in larger mammals. This reflects the fact that the heart rate is faster in smaller mammals than in larger mammals. Steiger (1977) showed that the rate constant of oscillatory work correlates well with the heart rate; *r*_4_ and *r*_−4_ are the determinants of the oscillatory work. The difference in the rate constants between animals may stem from the difference in the myosin heavy chain (MHC) isoform distributions. It has been known that ventricles of larger mammals (e.g., bovine, human) carry β-MHC, whereas those of smaller mammals (e.g., mouse, rat) carry α-MHC. We showed that rats papillary muscles carrying α-MHC have faster *r*_4_ and *r*_−4_ than those carrying β-MHC (induced by propylthiouracil), which have slower *r*_4_ and *r*_−4_ (Table 3) (Kawai et al. 2016). This difference may stem from the MHC’s sequence difference in the segment 617–638. Another sequence difference in the segment 793–814 that attaches MLC, and that may act as a break for actomyosin interaction (Wang et al. 2013), because of the difference in ATP binding step *K*_1_ and CB detachment step *r*_2_ and *r*_−2_ (Kawai et al. 2016). It has been known that the ATP hydrolysis rate is faster in α-MHC than β-MHC (Deacon et al. 2012; Michael et al. 2014; Michael and Chandra 2016). The differences between the two myosin isoforms in the kinetic constants have been observed across multiple species (Millar and Geeves 1988; Ritchie et al. 1993; Bloemink et al. 2007).

#### 3.1.4. CB occupancy at each state

From these kinetic constants, the CB probability (occupancy) at each state (*X*_4_, *X*_5_, and *X*_6_) is calculated as the function of *P* = [Pi] by using the Mass Action Law (*X*_5_ = *K*_4_*X*_4_, *X*_6_ = *X*_5_/*K*_5_*P*) with a constraint of: *X*_4_ + *X*_5_ + *X*_6_ = 1.

2 $${X}_{4}=\frac{1}{M},$$

3 $${X}_{5}=\frac{{K}_{4}}{M},$$

4 $${X}_{6}=\frac{{K}_{4}}{({K}_{5}P)M},$$

5 $$\text{where}\, M\equiv 1{+K}_{4}+\frac{{K}_{4}}{{K}_{5}P}=1+\frac{{K}_{4}(1+{K}_{5}P)}{{K}_{5}P}.$$

In Fig. 2B, *X*_4_, *X*_5_, *X*_6_, and *X*_5_ + *X*_6_ are plotted as functions of *P*. As expected, *X*_4_ and *X*_5_ are increasing functions of *P*, and *X*_6_ and *X*_5_ + *X*_6_ are decreasing functions of *P*. Steady state force (*F*_1_) is also measured before and after sinusoidal analyses, averaged, and studied as the function of *P*: *F*_1_ = *F*_1_(*P*). This is plotted in Fig. 2C (discrete points). As has been found by many investigators (Pate and Cooke 1989; Millar and Homsher 1990; Potma et al. 1995; Wahr et al. 1997; Tesi et al. 2002a), force is a decreasing and saturating function of *P*. Force supported by each CB state (*T*_4_, *T*_5_, and *T*_6_) is deduced based on the assumption that CBs are arranged in parallel in the half sarcomere, and that their force is additive (Kawai and Zhao 1993):

6 $$F_1(F)=T_4X_4+F_5X_5+F_6X_6=\frac{{K}_{4}}{M}{T}_{5}+\frac{{K}_{4}}{({K}_{5}P)M}{T}_{6},$$

7 $${F}_{1}\left(P\right)=\frac{{K}_{4}\left(1+{K}_{5}P\right)}{\left({K}_{5}P\right)M}{T}_{5 }\,\quad\left(\text{if }\,\,{ T}_{6}={T}_{5}\right),$$

8 $${\text{In}}\;{\text{particular}},\;F_{1} \left( 0 \right) \, = T_{6} ,$$

9 $${F}_{1}\left(1/{K}_{5}\right)=\frac{{K}_{4}}{1+{2K}_{4}}\left({ T}_{5}+{T}_{6}\right),$$

10 $${F}_{1}\left(\infty \right)=\frac{{K}_{4}}{1+{K}_{4}}{ T}_{5},$$

where *T*_4_ is the force supported by *X*_4_; *T*_5_ is the force supported by *X*_5_, and *T*_6_ is the force supported by *X*_6_. We assume that* X*_4_ does not carry any force (*T*_4_ = 0), because this is the state before force generation. By fitting the data of force vs. *P* (Fig. 2C) to Eq. 6, using the data for *K*_4_ and *K*_5_ obtained from the rate constant study (Eq. 1 and Fig. 2A), we obtain *T*_5_ and *T*_6_. This is a standard linear fitting with two fitting parameters; hence, *T*_5_ and *T*_6_ can be readily deduced together with their confidence ranges. It turned out that *T*_5_≈*T*_6_ gives the best fit (Kawai and Halvorson 1991; Kawai and Zhao 1993; Wang and Kawai 1997; Kawai et al. 2018; Kawai and Iorga 2024), indicating that force generation occurs before Pi release, and that the same force is maintained after Pi release. Here *N*_DF_ = 52 (if *T*_5_ = *T*_6_) and is large. Notice that *X*_5_ + *X*_6_ (Fig. 2B) provides a good match to the force vs. [Pi] plot (Fig. 2C). However, *X*_6_ alone does not fit the force data in Fig. 2C at all, suggesting that force generation does not occur after Pi release. The data in Fig. 2 were reproduced from Wang and Kawai (1997) in rabbit soleus slow-twitch fibers. The same was found to be the case for mouse soleus slow-twitch fibers (Kawai et al. 2018), for rabbit psoas fast-twitch fibers (Kawai and Halvorson 1991; Kawai and Zhao 1993), and in single myofibrils from rabbit psoas (Kawai and Iorga 2024).

#### 3.1.5. Identification of CB states

In Fig. 1B, we can then identify that *X*_6_ is the state after Pi release; hence, it is the strongly attached AM*ADP state.* X*_5_ is the state before Pi release with force; hence, it is the strongly attached AM*ADP.Pi state. *X*_4_ is the state before force generation; it can be either the detached state M.ADP.Pi, the weakly attached state AM.ADP.Pi, or the strongly attached state AM.ADP.Pi without force. These are depicted in Fig. 1C. Here the “weakly attached state” may mean the ionic interaction between actin and myosin, but so far no stable intermediate state has been identified. The strongly attached state indicates stereospecific and hydrophobic interactions between actin and myosin (Rayment et al. 1993a). In principle, the strongly attached AM.ADP.Pi state without force is essential for force generation, but this could be a short-lived transient state. Its brief existence was recently demonstrated in an experiment using cryo-electron microscopy (EM; Klebl et al. 2025). As soon as the no-force AM.ADP.Pi state is formed, it quickly goes to Step 4, as shown in the myosin V construct (Trivedi et al. 2015). The above analyses, based on fiber and myofibril experiments, concluded that force is generated in Step 4 before Pi is released, and the same force is maintained with Pi release. This conclusion is a direct interpretation of the data with statistics, based on a simple three-state model (Fig. 1B/C); hence, its confidence level is very high (less than ± 15% of the value).

Isometric force as a function of [Pi] has been measured by many investigators, including (Rüegg et al. 1971; Cooke and Pate 1985; Kawai 1986; Godt and Nosek 1989; Millar and Homsher 1990; Potma et al. 1995; Regnier et al. 1995; Wahr et al. 1997) to name a few. A question that one might ask is, if the data of force vs.* P* is fitted to Eq. 6 or 7 directly, without knowing the kinetic constants, could 3–4 parameters (*K*_4_, *K*_5_, *T*_5_, and *T*_6_) be deduced? A quick look at Eq. 6 shows that as *P* → 0, force becomes *T*_6_ (Eq. 8); hence, *T*_6_ could be determined independently. One difficulty with this approach is that Eq. 6 is an approximation, and the approximation becomes worse as *P* → 0 because Step 6 then becomes more significant, which repopulates X_4_ and X_5_. Therefore, in this case *T*_6_ is not as reliable. Another difficulty is Pi contamination and its continuous production as a result of ATP hydrolysis (Sect. 3.6). Equation 6 shows that as* P* → ∞, force asymptotically approaches *T*_5_*K*_4_/(1 + *K*_4_) (Eq. 10); hence, this term can be determined, and if *T*_5_ = *T*_6_ is assumed, then *K*_4_ can also be determined. A method similar to this was tried by Tesi et al. (2002a). In their analysis, they used their kinetic results at two different [Pi] (0 mM and 5 mM) on *k*_Pi(+)_ and combined these results with earlier data on fast-twitch fibers published by Dantzig et al. (1992), Kawai and Zhao (1993), Regnier et al. (1995), and Wahr et al. (1997) and on slow-twitch fibers published by Millar and Homsher (1992), Wahr et al. (1997), and Wang and Kawai (1997) to get the results shown in Tables 2 and 3. This effort concluded that force generation occurs before Pi release.

As shown above, it would be desirable to carry out kinetic analysis first, determine the rate constants of Step 4 (*r*_4_ and *r*_−4_) and the Pi association constant (*K*_5_), establish a CB scheme, and then correlate the results with force measurements to determine *T*_6_ (force supported by the AM*ADP state) and *T*_5_ (force supported by the AM*ADP.Pi state). It is also important to determine 95% confidence limits of these parameters, or at least the SEM values of the parameters obtained (see Tables 2 and 3 for the presence of statistical analyses, under the ± column). When the data are scattered, as discussed by Tesi et al. (2002a), the confidence ranges become large. As correctly pointed out by these authors, one reason for scatter is Pi contamination, which becomes worse in the core of fibers during maximal activation. One solution is to determine Pi contamination or to reduce this (Sect. 3.6).

### 3.2. Experiments performed during force decrease

Force decrease happens when [Pi] is increased suddenly by photolysis of caged Pi, after a solution switch, or when Ca^2+^ is removed from the activating solution to relax the fibers/fibrils.

#### 3.2.1. Caged phosphate experiments in fibers

Dantzig et al. (1992) used caged Pi (1-(2-nitrophenyl)ethylphosphate), which itself is inactive in the actomyosin system. They soaked rabbit psoas fibers in an activating solution with caged Pi, illuminated the fibers with UV light (wavelength 320 nm), and released Pi at a fast rate (2.4 × 10^4^ s^−1^). This experiment is called the “Pi-jump.” After a short pause (~ 2 ms; this varied with [Pi]), force decayed exponentially, and its rate constant was measured. They plotted [Pi] vs. the rate constant, which is similar to that in Fig. 2A, and deduced the CB scheme shown in Fig. 1B/C. During a short pause, Pi is bound to actomyosin, resulting in the AM*ADP.Pi state, followed by a transition to AM.ADP.Pi, which does not have force (Dantzig et al. 1992). Thus, their interpretation of the results is the same as ours (Kawai and Halvorson 1991). Because the amount of Pi released by a single UV flash is limited to 3 mM, their earlier results covered [Pi] in the range of 0–3 mM Pi (or 2–4 mM including contamination), from which they characterized the rising phase of [Pi] vs*.* rate constant plot (Millar and Homsher 1992); Fig. 2A looks linear when [Pi] ≤ 4 mM. But their later results covered a larger concentration range by using a step-wise increase in [Pi] in the activating solution and liberation of Pi from caged Pi (Dantzig et al. 1992). By using caged Pi on rabbit psoas fibers, Walker et al. (1992) had similar results. These results are compared in Table 2.

#### 3.2.2. Sudden increase in [Pi] in single myofibrils

Pi-jump experiments cannot be performed by a simple solution change in fibers (50–100 μm in diameter), because it takes a few seconds until their cross section is equilibrated with the new [Pi], and because the elementary steps being studied take place in the order of 10 ms. However, in single myofibrils (1–2 μm in diameter), the tension time course can be studied with a solution change (called “solution switch”), because the solution in myofibrils can be changed within the time constant of 2 ms (Tesi et al. 2002a) or even in 1 ms (Stehle 2017). Using rabbit psoas single myofibrils, Tesi et al. (2000) switched the activating solution from 0.1 mM [Pi] to 5 mM [Pi] at 5°C. With this solution switch, force decayed exponentially, and they observed a rate constant of 18.2 s^−1^. With Q_10_ = 1.6 (Q_15_ = 2.0) of *r*_−4_ (Zhao and Kawai 1994), this rate constant becomes 36 s^−1^ at 20°C, which is comparable to the other values (Table 2).

#### 3.2.3. Sarcomere collapse (give)

An interesting phenomenon was noticed in single myofibril experiments. During fast exponential force relaxation (*k*_REL_), Stehle et al. (2002), Poggesi et al. (2005), and Stehle (2017) observed that one sarcomere in the middle suddenly relaxed, and this relaxation propagated to neighboring sarcomeres, spreading the relaxation. Tesi et al. (2002b) called this “sarcomere give/collapse.” The mechanism that leads to this phenomenon is well explained by Poggesi et al. (2005). In earlier studies, an increase in sarcomere dispersion was observed during relaxation using optical diffraction studies in intact frog single muscle fibers (Kawai and Kuntz 1973; Edman 1980; Edman and Flitney 1982). This dispersion increase may happen when force decreases, such as when [Pi] is increased (termed as “sarcomere give” by Stehle 2017), which may cause a problem in our interpretation of the results. This is because the basic assumption of fiber and/or myofibril studies is that every sarcomere behaves in the same way. Consequently, the question asked here is: does the sequential sarcomere relaxation obscure the rate constant measurements? This is a less than ideal situation, but the results of Tesi et al. (2000) are comparable to other isometric experiments during steady state (Table 2), which may validate the interpretation. Under isometric condition with sinusoidal analysis, the sarcomere give did not happen in single myofibril experiments from rabbit psoas (Iorga et al. 2012; Kawai et al. 2021).

### 3.3. Experiments performed during force increase

An increase in force happens after a length release of active muscle fibers, a release and restretch to measure *k*_TR_, a sudden temperature increase, a decrease in [Pi], or during Ca^2+^ activation. In the following discussion, we only consider cases when diffusion does not limit the time course.

#### 3.3.1. The pressure release experiments

Pressure release experiments were carried out by Geeves’ group (Fortune et al. 1991). Under high pressure, fibers were activated, and the pressure was released suddenly, which resulted in a further exponential rise in tension (~ 17%). They plotted [Pi] *vs.* the rate constant of the rise and arrived at the same CB scheme as shown in Fig. 1B/C, with the same conclusion: force develops before Pi is released, and the same force is maintained after Pi is released. Their rate constant of “Phase 2” (our *r*_4_) is about 17 s^−1^ at 12 °C. Its adjusted value at 20 °C is 62 s^−1^ (Table 2), which is comparable to results of sinusoidal analysis and caged Pi experiments (Table 2).

#### 3.3.2. Unloaded shortening experiments

Caremani et al. (2008) performed an unloaded shortening experiment in rabbit psoas fibers at 12°C, in which an isometrically contracting muscle length was released by 3%, and the Pi effect on force redevelopment was studied. They noticed two exponentials (fast and slow); the rate constant of the fast exponential (*r*_F_) increased with [Pi]. They interpreted their results with the CB scheme in Fig. 1B/C and deduced the kinetic constants (listed in Table 2). The temperature adjusted *r*_4_ to 20°C is comparable to other reports (Table 2), but the Pi dissociation constant was large (1/*K*_5_ = 28.9 mM), because the [Pi] vs. *r*_F_ plot was almost linear with a slight curvature. The polarity of the amplitudes of their fast and slow exponentials were the same, presumably because of the large length release (3%). The polarity is opposite for a smaller length release or stretch, i.e., ~ 0.2% (Huxley 1974; Abbott and Steiger 1977; Kawai 1986).

#### 3.3.3. *k*_TR_ measurements

Quick length release and re-stretch experiment to measure *k*_TR_ (rate constant of tension redevelopment) was introduced by Brenner and Eisenberg (1986) and Brenner (1988), and this method was used by Wahr et al. (1997) to study the Pi effect. They showed the hyperbolic saturation curve of [Pi] *vs*. *k*_tr_ plot, similar to Fig. 2A. In rabbit psoas fibers, they found *r*_4_ = 12 s^−1^ at 15°C (Table 2). With the temperature adjustment to 20°C, *r*_4_ becomes 26 s^−1^ which is 2–3 × less than that in sinusoidal analysis or caged Pi results (Table 2), presumably because *k*_tr_ is influenced by a slower step in the CB cycle (discussed in Sect. 3.3.5. Their *K*_4_ and *K*_5_ are comparable to other measurements. Regnier et al. (1995) also used the *k*_TR_ analysis and studied the Pi effect in the range 0–10 mM Pi in rabbit psoas fibers, but their plot showed nearly a linear relationship, in contrast to the caged Pi results of Dantzig et al. (1992) or the plot in Fig. 2A. Stehle (2017) reported a linear relationship between [Pi] and the rate course of force decline when [Pi] was increased in the range 0–20 mM in guinea pig ventricular single myofibrils. He observed the same relationship when *k*_TR_ was measured in the same [Pi] range. During the force decline, he observed sarcomere give, and he interpreted these time course as that of the rate limiting step which cannot be separated from the force generating step.

#### 3.3.4. Temperature jump experiments

It is known that isometric force increases as the ambient temperature is raised in vertebrate muscles (Goldman et al. 1987; Rall and Woledge 1990), implying that the force generation step is an endothermic reaction (absorbs heat). Consequently, in the hope of elucidating the mechanisms of force generation, experimental devices have been constructed to increase the temperature quickly (e.g., Davis and Harrington 1987; Goldman et al. 1987). These experiments are called temperature jump (T-jump) experiments, and performed on muscle fibers.

In experiments carried out by Ranatunga (1999a), rabbit psoas fibers were activated at 9°C, a steady state in tension was reached, and the temperature was suddenly increased to 12°C by a laser pulse. Force was increased in two phases (Phases 1 and 2; Table 1); the faster Phase 1 was Pi sensitive. The force increase was 25‒47%, depending on [Pi]. Ranatunga plotted [Pi] *vs.* the rate constant of the increase. The plot was a saturating hyperbola, similar to Fig. 2A, and arrived at the same CB scheme as shown in Fig. 1B/C. From this, he concluded that force develops before Pi release. The rate constants he observed at 12°C were *r*_4_ = 49.5 s^−1^ and *r*_*−*4_ = 135 s^−1^, which are large and are comparable to other reports at 20°C. The reason for the large values is unknown, but it is possible that T-jump affects other faster steps in the CB cycle to influence the results.

**Fig. 3:**
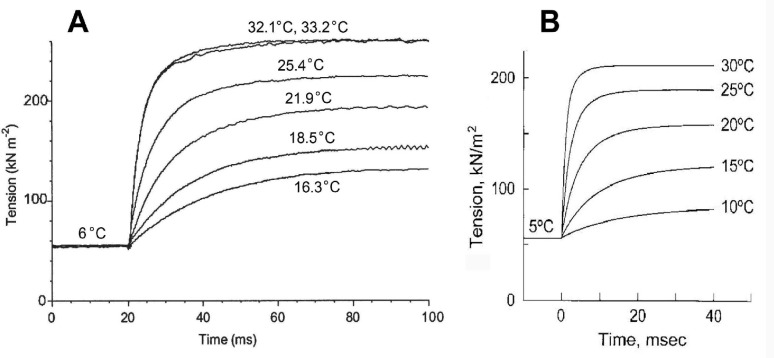
Temperature jump study in rabbit psoas fibers.** A** Data from Bershitsky and Tsaturyan (2002), reproduced and relettered with permission from *Journal of Physiology* (John Wiley & Sons). **B** Simulation from Kawai (2003) based on kinetic constants observed by Zhao and Kawai (1994). The abscissa has been shrunken, and the ordinate has been expanded to match **A**

A series of T-jump experiments were carried out by Bershitsky and Tsaturyan (2002), and their results are reproduced in Fig. 3A. Rabbit psoas fibers were activated at 6°C, force developed, and when a steady state was reached, they increased the temperature to various levels between 16.3 and 33.2°C. They did not study the effect of Pi.

Because sinusoidal analysis can characterize the elementary steps of the CB cycle at a fixed temperature under the isometric condition, we studied all kinetic constants of elementary steps at six fixed temperatures, in 5 °C increments between 5 and 30 °C (Zhao and Kawai 1994), which were plotted in Figs. 5 and 8 of their paper. Q_10_ of their kinetic constants are listed in Table 4.

**Table 4:** Temperature sensitivity of the kinetic constants in rabbit psoas fibers with sinusoidal analysis (Zhao and Kawai 1994)

Kinetic constant	Elementary step	Value at 20 °C	Q_10_	Temperature range (°C)
Equilibrium constants				
*K*_0_	ADP binding	2.3 ± 0.2 mM^−1^	0.3	10–20
			1.2	20–30
*K*_1_	ATP binding	0.43 ± 0.06 mM^−1^	0.12	10–20
			1.1	20–30
*K*_2_	CB detachment	1.77 ± 0.16	0.9	10–25
*K*_4_	Force generation	2.16 ± 0.11	4.3	10–25
*K*_5_	Pi binding	0.12 ± 0.02 mM^−1^	1.1	10–25
Rate constants				
*r*_2_	CB detachment	250 ± 20 s^−1^	2.6	10–25
*r*_−2_	Reverse detachment	143 ± 7 s^−1^	3.0	10–25
*r*_4_	Force generation	161 ± 5 s^−1^	6.8	10–25
*r*_−4_	Reverse force generation	77 ± 5 s^−1^	1.6	10–25
*r*_6_	ADP isomerization	16 ± 1 s^−1^	1.1	10–25

As seen in Table 4, kinetic constants have very different Q_10_s of large ranges (rate constants 1.1–6.8, equilibrium constants 0.12–4.3), hence, the temperature effect cannot be generalized. One may notice that Step 4 (force generation) has the largest Q_10_ on the rate constant *r*_4_ (Q_10_ = 6.8), followed by its equilibrium constant *K*_4_ (Q_10_ = 4.3), but the reversal step *r*_−4_ is not as temperature sensitive (Q_10_ = 1.6). Dantzig et al. (1992) observed similar results with caged Pi: Q_10_ for *r*_4_ was 3.34, for *K*_4_ was 2.5, and for *r*_−4_ was 1.4. These results explain the large temperature effect on isometric force (Fig. 3). Quite interestingly, the temperature sensitivity of isometric force among vertebrate skeletal muscles increases as animals advance along the evolutionary tree, from earliest to most recent: icefish < bullrout < frog < iguana < mouse and human (Rall and Woledge 1990).

Because we know (a) the kinetic constants for all steps in the CB cycle at 5 °C intervals between 5 and 30 °C (Zhao and Kawai 1994); (b) how to calculate CB distributions based on the kinetic constants (as in Eqs. 2–5); (c) how much force each CB state supports (Fig. 7); and (d) how to calculate the total force generated (as in Eq. 6 or 7), the active force development of T-jump experiments can be simulated. These data were obtained by sinusoidal analysis together with Pi, ATP, and ADP effects on the apparent rate constants, to deduce the rate constants of elementary steps at six different temperatures ranging between 5 and 30 °C. *r*_6_ was obtained from the ATP hydrolysis measurements at six different temperatures and as described in Eq. 18 later. Results of this simulation are shown in Fig. 3B (Kawai 2003). A comparison between the data (Fig. 3A) and simulation (Fig. 3B) demonstrates that both sets of time courses look like exponentials and are strikingly similar. This good match demonstrates the correctness of the generally held assumption that force/CB remains the same as temperature is raised, and that a larger number of force-generating CBs are formed, which results in higher force (Zhao and Kawai 1994; Kawai 2003).

Working with intact frog tibialis anterior fibers, Piazzesi et al. (2003) and Decostre et al. (2005) observed little change in stiffness but increased force with temperature. They interpreted their results that force/CB increases, but to a lesser degree, the number of force generating CBs increases. However, these measurements are difficult to evaluate because there were no descriptions on the temperature sensitivity of the force generating step, measurement of rate constants, and not much evidence was given to indicate what was measured by stiffness (weakly attached, strongly attached with/without force, viscosity). Their major interpretation is not consistent with the Dantzig et al. (1992)’s finding that the force generation step (*r*_4_) has a large Q_10_ of 3.34 (see also Table 4), which predicts that significantly more force generating CBs are attached at higher temperatures. Neither is their interpretation supported by the results of in vitro motility assay (Kawai et al. 2006), in which we counted the number of attached CBs. The latter study demonstrated that the number of force generating CBs increases with the temperature in the range of 20–35 °C, and concluded that force/CB remains the same using proteins purified from rabbit white skeletal muscles.

On closer look at Fig. 3, there is a subtle difference in the rates of tension development. At high temperatures (25–33 °C), the time course appears to be faster during the simulation (Fig. 3B) than during experimental observation (Fig. 3A), but this difference becomes less apparent at low temperatures (15–20 °C). Why does this happen? The difference in the two measurements can be explained by the fact that the sinusoidal analysis was carried out when force was at a plateau and steady, whereas the T-jump experiments were carried out while force was increasing. The force increase requires a stretch of a series elastic element(s), which requires CBs to cycle, as discussed in Sect. 3.3.5. This may cause a delay in force development. The larger the force increase is (jump to a higher temperature), CB needs to stretch series elastic elements further to generate a larger force, which requires more CB cycles that include a slow (rate-limiting) step. Consequently, the results of the T-jump experiments are slower than those of the simulation, in particular at higher temperatures. This may provide an answer to the question by Offer and Ranatunga (2015): why are T-jump results slower than length steps? Sinusoidal analysis is a frequency domain version of the length-step experiment.

A shortfall of the T-jump experiments is that temperature affects practically every chemical reaction, which is also true in the elementary steps of the CB cycle, as shown in Table 4. Consequently, it is very difficult to characterize a specific elementary step in the CB cycle. Another difficulty is that an increase in force is slow (Sect. 3.3.5). 

#### 3.3.5. Why is the time course slow when force increases?

A force increase happens after a length release (± restretch), T-jump, decrease in [Pi], during Ca^2+^ activation (*k*_act_), etc. The time course of these experiments is slow, in particular when the increase of tension is large. One possible way to explain this slow increase is that because series elastic elements are slack (no tension) during relaxation, this slack must be removed for the force to be detected. Series elastic elements include thick and thin filaments, titin (connectin), CB, Z-line, M-line, and nebulin and tendon (if present). Removal of the slack requires CBs to cycle at least once and likely more. This makes the time course slow, because there is a slow step in the CB cycle. Transient analysis (simple examples are shown in Appendices 1–3) is based on a perturbation of equilibria, and it measures faster rate constants, but it does not measure the slowest rate constant. A fast apparent rate constant can be approximated by a linear combination (sum) of back and forward intrinsic rate constants (Eq. 1; other examples are Eqs. 16, 17, A4, and A30), but the analysis of the rate-limiting step (smallest rate constant) needs a different approach. For this goal, we proposed a model that considers the rate-limiting step (Wang and Kawai 2013). In this model, the total CB cycle time (*τ*) is the sum of fractions of the time CB spends at each elementary step:

11 $$\tau = \tau_{2} + \tau_{4} + \tau_{6} \approx \tau_{4} + \tau_{6} ,$$

12 $${\text{where}}\;\tau \equiv 1/\nu ,\quad \tau_{2} \equiv 1/\nu_{2} ,\quad \tau_{4} \equiv 1/\nu_{4} ,\quad {\text{and}}\quad \tau_{6} \equiv 1/\nu_{6}$$

and *τ*_i_ is the average time CBs spend at Step *i* (*i* = 1, 2,…, 6), which is the reciprocal of the apparent rate constant *ν*_i_ (Eq. 12). Steps 1, 3, 3a, and 5 are fast (approximated by equilibria), hence they drop out from Eq. 11 (*τ*_1_ ≈ *τ*_3_ ≈ *τ*_3a_ ≈ *τ*_5_ ≈ 0). During isometric contraction, we identified the slowest step as Step 6 (isomerization of ADP bound state), followed by ADP release (AM*ADP → AM.ADP ↔ AM + ADP) (Kawai and Halvorson 1991; Zhao and Kawai 1994), with *r*_6_ = 16 s^−1^ (or *τ*_6_ = 63 ms) in rabbit psoas at 20°C (Sect. 6.2). Therefore, *ν*_6_ = *r*_6_, because Step 6 is almost unidirectional. *ν*_4_ is the same as 2π*b* and depends on [Pi], as shown in Eq. 1. *ν*_2_ is the same as 2π*c* and depends on [MgATP] and [MgADP] (Kawai 1978; Kawai and Halvorson 1989). Because *ν*_2_ > *ν*_4_ > *ν*_6_ (Step 2 is faster than Step 4, which is faster than Step 6), then *τ*_2_ < *τ*_4_ < *τ*_6_, so that *τ*_2_ is the least important term in Eq. 11; hence it is removed for simplicity. *ν* (= 1/*τ*) is the number of cycles CBs complete in 1 s, hence it is the “turn-over rate.” Eq. 11 is useful in analyzing the rate-limiting step, which cannot be analyzed by the perturbation analysis method. From Eqs. 11 and 12,

13 $$\frac{1}{\nu }=\frac{1}{{\nu }_{4}}+\frac{1}{{r}_{6} }\,\, \text{ or  }\,\, \nu =\frac{{\nu }_{4}{r}_{6}}{{\nu }_{4}+{r}_{6}}.$$

An example of Eq. 13 is the ATP turnover rate (Eq. 18), which is limited by the slowest step.

Another example is the force development time course with the rate constant *k*. According to the theory developed by Wang and Kawai (2013) (their Eq. 21),

14 $$k=\alpha \nu =\frac{\alpha }{\frac{1}{{\nu }_{4}} + \frac{1}{{r}_{6}}}=\frac{\alpha {\nu }_{4}{r}_{6}}{{\nu }_{4}+{r}_{6}},$$

15 $$\text{where}\,\alpha \equiv \frac{{\sigma \eta }_{0}\left(\frac{1}{\lambda }-1\right)}{{F}_{\text{stp}}}.$$

$\sigma$ is series elasticity, ${\eta }_{0}$ is the step size, *λ* is the ratio of isometric to unloaded cycling rates (*λ* < 1), and *F*_stp_ is force when CB stepping stops (Wang and Kawai 2013). The effect of [Pi] on *k* is via *ν*_4_ (Eq. 14): *ν*_4_ (= 2π*b*) increases with [Pi] (Eq. 1, Fig. 2A), and so does *ν* (Eq. 13). *α* is a constant.

Unfortunately, this model was misunderstood by Stehle and Tesi (2017). They set

16 $$k_{{{\text{TR}}}} = f_{{{\text{app}}}} + g_{{{\text{app}}}} + f_{{{\text{app}}}}^{ - }$$

and equated our *r*_6_ (rate-limiting step) to their *g*_app_. *k*_TR_ is the rate constant of force redevelopment time course, after a quick release by 10–20% and a restretch to the original length after 20–50 ms (Brenner and Eisenberg 1986; Brenner 1988). Stehle and Tesi (2017) stated that a small *g*_app_ cannot limit *k*_TR_ according to Eq. 16, suggesting that our model was wrong. The conclusion of our model was that the slow rate of *k*_TR_ cannot be explained by Eq. 16, but it can be explained by Eq. 14. Equation 16 was originally developed by Brenner (1988). As described in Appendix 1, Eq. 16 can explain a fast rate constant, but not the rate-limiting step. It turned out that the *k*_TR_ that has been observed is < 40 s^−1^, but the rate constants of elementary steps are > 70 s^−1^ (Table 4); see also Heinl et al. (1974), Kawai (1978), Goldman et al. (1984), and Goldman and Simmons (1984), with some reaching 1000 s^−1^ (Huxley and Simmons 1971).

Equation 16 can be readily derived based on the two-state model of Fig. 1A as described in section A1.3 (Eq. A20). This model’s basic assumption is a perturbation of an equilibrium shown in Fig. 1A. The apparent rate constant is ruled by a fast reaction, a slow reaction is less sensitive, and it does not consider or measure the rate-limiting (slowest) step. These were not explicitly stated in Brenner (1988), but his derivation shows that these were implicit assumptions.

Because *r*_4_ (hence *ν*_4_) is more sensitive to temperature (Q_10_ = 6.8) than *r*_6_ (Q_10_ = 1.1) (Table 4), temperature change profoundly affects their interrelationship, and so does the fiber type. *r*_6_ itself is measured separately from faster kinetic constants, as described in Sect. 6.2, and it can be a variable. The difference in the temperature sensitivity (Table 4) intricately affects Eq. 13 to give different impressions. These are the reasons why *k*_TR_ is somewhat sensitive to [Pi] depending on the conditions as mentioned by Stehle (2017) and Stehle and Tesi (2017). The analyses shown in Eqs. 11–15 are not limited to *k*_TR_, but they can be applied to a time course that accompanies a large force increase. *k*_act_ has a similar time course to *k*_TR_ (Tesi et al. 2002a); therefore, *k*_act_ can be analyzed in a similar way. This mechanism can explain why the kinetics of the Pi effect are slower when measured through *k*_TR_ (Wahr et al. 1997) than through other analysis methods (Table 2). Similarly, this mechanism can explain why a slow time course is observed in single myofibrils when [Pi] is decreased compared to when [Pi] is increased to the same concentration (Tesi et al. 2000, 2002a), depending on the condition as pointed out by Stehle (2017).

Another example of slow time course is T-jump experiments, which are slower than anticipated, as shown in Fig. 3 (Sect. 3.3.4). The experiment conducted by Ranatunga (1999a) (Table 2) did not have this problem, because the increase in temperature was only 3 °C with a fraction of force development. Pressure release experiments by Fortune et al. (1991) did not have this problem either. In those experiments, fibers were already in tension (series elastic elements were already stretched) before the perturbation was applied, and the time course was based on an increase in a small fraction of force. Consequently, if such experiment is planned, it would be desirable to perform experiments with partially activated fibers and induce a fractional force increase with a perturbation. Apparently, the smaller the increase, the more dependable the results will be.

It has been reported that increasing [Ca^2+^] increases *k*_TR_ in frog cardiomyocytes (Brandt et al. 1998) and in rabbit psoas fibers and in myofibrils (Kreutziger et al. 2008). These observations can be interpreted in the following way. In mouse cardiac fibers, it was found that 2π*b* (= *ν*_4_) increases proportionately to [Ca^2+^] at low Ca^2+^ concentrations (Xi et al. 2024). At this stage *ν*_4_ < *r*_6_ (*ν*_4_ = 0 during relaxation), hence *ν*_4_ (Step 4) limits the CB cycle, which is observed by *k*_TR_ according to Eq. 14 (*k*_TR_≈*αν*_4_). As [Ca^2+^] is increased, *ν*_4_ exceeds *r*_6_ (*ν*_4_ > *r*_6_), then thereafter *r*_6_ limits the CB cycle. Above all, *k*_TR_ increases with [Ca^2+^] and approaches saturation, as observed by Brandt et al. (1998) and Kreutziger et al. (2008).

### 3.4. Single molecule studies and stopped flow assays on labelled proteins

The results of single molecule studies are summarized in Table 5. Woody et al. (2019) studied the steps surrounding Pi release in single molecules from C2C12 myoblast synthesized human β-cardiac myosin (HMM construct of myosin II) and observed that an addition of 10 mM Pi did not alter the rate or the size of the power stroke, which was faster than the rate of Pi release (Table 5). The important point here is that Step 4 (*r*_4_) is faster (larger) than Step 5 (*r*_5_, Table 5); hence, force generation (cleft closure and tilting of the lever arm) occurs earlier than Pi release. This conclusion is consistent with the CB scheme shown in Fig. 1B/C, in which force generation occurs at Step 4. Woody et al. (2019) also concluded that the occupancy of CBs at the AM.ADP.Pi state is small and transient. This small and transient occupancy agrees with our results on single myofibrils (Kawai and Iorga 2024). The rate constants of the power stroke they measured were *r*_4_ = 1000 s^−1^ and *r*_−4_ = 250–1000 s^−1^. With a similar single molecule set up, Scott et al. (2021) studied myosin Va and actin filament interaction. They used myosin mutant S217A to slow the Pi release step and observed that the same size power stroke took place even with this mutation, indicating that the power stroke occurs before Pi is released (*r*_4_ > *r*_5_). They measured *r*_4_ ≥ 500 s^−1^.

**Table 5:** Results of single molecule studies and stopped flow assays

Authors	Myosin construct	*r*_4_	*r*_−4_	*r*_5_	±	Temperature
	*Purified from muscle	s^−1^	s^−1^	s^−1^		°C
*Single molecule studies*						
Woody et al. (2019)	Myosin II, β cardiac	1000	250‒1000	400	‒	20^##^
Scott et al. (2021)	Myosin-Va, S1	≥ 500		143	x	30
*Stopped flow assays*						
Trivedi et al. (2015)	Myosin V	352, 493		201	x	25
Muretta et al. (2015)	Myosin II, skeletal HMM	> 350		30–40	‒	25

*r*_4_ is the rate of the lever arm swing, and *r*_*−*4_ is its reversal (Fig. 1B). *r*_5_ is the rate of Pi release

^##^Temperature fromWoody et al. (2018)

The results of stopped flow assays are also included in Table 5. Placing a fluorescent probe on a Cys residue on calmodulin (CaM), which binds to the IQ motif of the lever arm of myosin V (construct had only one IQ motif), Trivedi et al. (2015) demonstrated that the lever arm swing (power stroke) occurs in two steps: (a) a fast step before Pi release and (b) a slow step before ADP release. This is achieved by using the Förster resonance energy transfer (FRET) technique with a single molecule assay on the myosin V construct. The fast step (*r*_4_) occurred at a rate of 352 ± 33 s^−1^ or 493 ± 119 s^−1^ (depending on the label), and the slow and smaller step occurred at a rate of 18 ± 9 s^−1^ or 20 ± 13 s^−1^. The first step corresponds to Step 4, and the slow step corresponds to Step 6 in Fig. 1. Trivedi et al. (2015) observed that the rate of the first step was twice the rate of Pi release (*r*_5_ = 201 s^−1^) and proposed that the lever arm swing (force generation) promotes Pi release. Muretta et al. (2015) used the same assay on an HMM of myosin II and observed that lever arm rotation is much faster than Pi release; hence, lever arm rotation must occur before Pi release. Scott et al. (2021) made a similar observation on a myosin Va construct. These conclusions agree with the conclusions based on fiber and myofibril experiments—force generation occurs before Pi release.

Although it was not clearly stated in above reports, it appears that the sequence of events (lever arm swing and Pi release) was measured from the time of actin and myosin contact, and their reciprocals were reported as the rate constants. If this is the case, *r*_5_ has a contribution from *r*_4_, hence its effect must be subtracted. If this operation is performed, *r*_5_ increases somewhat (Pi release becomes faster), but there is no overall change in the conclusion.

### 3.5. Comparison with physiological studies

It is good science that the order of appearance of force (or lever arm swing) and Pi release agree between physiological studies on fibers/myofibrils and on single molecule studies including stopped flow assays. The rate constant *r*_4_, measured with single molecule experiments (Table 5), is much faster than the values measured in skeletal or cardiac fibers (Table 3). This difference may stem from the vast differences in experimental materials (actin filament and myosin II HMM or myosin Va construct vs. structured muscle systems) and experimental conditions (dilute proteins in dilute solutions vs. closely-packed proteins with physiological solutions). Single molecule studies are carried out in a condition in which contractile proteins are dilute, myosin does not have a tail, and actin does not have Tpm or Tn with Ca^2+^; hence, single molecule studies would not have the kind of cooperativity that muscle fibers would have. Consequently, allosteric effects between macromolecules may be absent or may not be as strong in single molecules as in muscle fibers/myofibrils. It is possible that the allosteric effect tunes macromolecules to produce a slower motion, because it appears that the slower motion is essential to perform momentum transduction (Sect. 6.3). The ionic strength of the solutions used in single molecule studies is generally < 50 mM compared to about 200 mM in skinned fiber/myofibril studies. The ionic interaction takes place within the Debye length (ionic atmosphere) which becomes larger in lower ionic strength, resulting in a stronger ionic interaction (Wang et al. 2015). In fact, Veigel et al. (1998) observed that the rigor development time course was much faster in a low ionic strength solution (60 mM) than in a high ionic strength solution (230 mM) in mouse EDL fibers, due to increased electrostatic interaction between actin and myosin.

### 3.6. Pi contamination and the effective Pi concentration

When studying the effect of Pi, it has become apparent that Pi contamination in the activating solution needs to be considered (Tesi et al. 2002a); the contamination arises with phosphate compounds. Dantzig et al. (1992) measured contamination in their activating solution by using a colorimetric method and reported it to be 0.8 ± 0.07 mM. Tesi et al. (2002a) estimated the contamination in their activating solution to be 0.17 mM, which can be reduced to < 0.05 mM by passing the solution through a column containing purine nucleoside phosphorylase. We determined the effective Pi contamination empirically by plotting Pi vs*.* 2p*b* × *B* (the apparent rate constant times the magnitude of Process B), which is proportional to the flux (rate) of the reversal of Step 4, and by extrapolating the plot 2p*b* × *B* → 0 on the negative Pi axis. This extrapolation found contamination to be 0.62 ± 0.08 mM in rabbit psoas fibers during maximal activation at 20°C (Kawai and Halvorson 1991). This is an averaged value across the cross section of fibers, because the Pi gradient is formed due to a continuous production of Pi as a result of ATP (actually, creatine phosphate) hydrolysis, which diffuses out from the fibers. This condition was analyzed by Cooke and Pate (1985).

The large diameter of muscle fibers (50–100 μm) does not limit our ability to measure rate constants stemming from CBs. All solution components are equilibrated and buffered before and during measurements that are carried out at the steady state. This is contrary to concerns of Debold (2021). Experiments are performed in the presence of creatine kinase and creatine phosphate (CP, or an equivalent enzyme coupled buffer system), so that ATP and ADP concentrations are maintained; only CP and creatine concentrations change, as ATP is used by CBs. However, CP and creatine are inactive elements and do not affect CB functions. Since Pi diffuses out from the fibers, a Pi gradient is formed across the fiber cross section. The effective difference in [Pi] was experimentally determined to be 0.62 ± 0.08 mM as described above; hence, the origin of Fig. 2 must be shifted to the left by this amount. But this does not make a big difference in the results or conclusions, and we have actually corrected for this difference (Kawai and Halvorson 1991). More recent experiments using single myofibrils (1–2 μm in diameter) demonstrate that qualitatively similar results are obtained in both fiber and myofibril systems, and it was concluded that in both systems force develops before Pi release (Table 2) (Kawai and Iorga 2024). Myofibrils do not have the diffusion problem that fibers do as described by Stehle (2017).

### 3.7. Other evidence of force generation before Pi release

Another piece of evidence on the force generation step comes from oscillatory work; oscillatory work and Process B of sinusoidal analysis are synonymous and describe the same phenomenon. As shown by Abbott (1972), oscillatory work is generated by repetitive transition of CBs between the force state and the non-force state. Our experiments have demonstrated that oscillatory work increases as [Pi] is increased (Kawai 1986; Kawai and Halvorson 1991). Conversely, if [Pi] → 0, oscillatory work diminishes and disappears, as shown in single myofibril experiments (Kawai et al. 2021; Kawai and Iorga 2024). These experiments demonstrate that the P_i_-attached state(s) of CBs is essential for oscillatory work production. This idea is consistent with Fig. 1B, where X_5_ is the state with force, and oscillatory work is produced as a result of X_4_‒X_5_ interconversion; consequently, force must be generated at Step 4. If force is generated at Step 5 after Pi release, then *X*_5_ does not have force; hence, oscillatory work could not be produced. If oscillatory work is interpreted by the CB scheme in Fig. 1A (i.e., no X_5_), then the observed rate constant (2π*b*) should increase linearly with [Pi], as shown by Eq. A19, which is contradicted by the data shown in Fig. 2A; see also Kawai and Iorga (2024). All the reports in Tables 2 and 3 showed saturating curves, similar to Fig. 2A. Oscillatory work is the mechanism that allows insects to fly by beating their wings (Pringle 1967), and it has been observed in skeletal muscle fibers (Kawai and Brandt 1980; Kawai and Schachat 1984; Wang and Kawai 1997) and in cardiac muscle fibers (Steiger 1977; Shibata et al. 1987; Saeki et al. 1991; Kawai et al. 1993; Wannenburg et al. 2000), implying that it is an essential feature of all striated muscle types and their contraction. Oscillatory work appeared to increase by partially (~ 20%) crosslinking CBs in rabbit psoas fibers to the level of insect fibers (Tawada and Kawai 1990); this increase can be explained by stabilization of sarcomeres which may decrease *r*_6_ (rate limiting step becomes slower).

## 4. Experiments performed during isotonic shortening

### 4.1. Force–velocity measurements

The results of Cooke et al. (1988), Caremani et al. (2013), and Månsson (2021) demonstrated that the maximum (unloaded) shortening velocity (*V*_0_) is not affected by [Pi], although isometric force (at *V* = 0) is affected by Pi. Therefore, it can be concluded that *V*_0_ does not contain information on steps involved in Pi release, and we cannot characterize the force generation step based on *V*_0_ measurement. Then the question is why does the effect of Pi disappear toward the unloaded condition? It is possible that as step 6 (*r*_6_) becomes faster (Sect. 6.2), the occupancy of state X_6_ (AM*ADP) reduces so the state that Pi binds becomes less available, hence there is little effect of Pi at the unloaded condition.

One may ask, “Could it be resolved with length transient?” The length transient was initially observed by Podolsky (1960) on frog Sartorius muscles with electrical stimulation. Caremani et al. (2013) observed Phases 2 and 3 in length transients, and these approximately correlate to Phases 2 and 3, respectively, of the tension transients reported by Huxley and Simmons (1971), Heinl et al. (1974), and Huxley (1974) in response to a step length change. However, their correlation is only approximate and not exact, and it is more empirical than theoretical. According to Caremani et al. (2013), Phase 2 of the length transient is not affected by [Pi], implying that Phase 2 does not carry information in the force generation and Pi release steps. Phase 3 was briefer, with an increased lengthening rate with Pi, but it appears that the signal was not strong enough to extract a rate constant and to characterize elementary steps. These imply that isotonic experiments and length transients are not suitable for characterizing steps surrounding Pi release and force generation in the CB cycle, although they may be useful to study the slowest (rate-limiting) step of the CB cycle. Incidentally, approximately Phase 2 of length transient corresponds to Process C of sinusoidal analysis, Phase 3 corresponds to Process B, and Phase 4 (shortening with the constant velocity) corresponds to Process A (Table 1). The fact that Process B is most sensitive to [Pi] (Kawai 1986) is consistent with observations by Caremani et al. (2013), except that the Pi effect is not as conspicuous in length transient results as it is in sinusoidal analysis results on force transients.

One note is that Pate and Cooke (1989) observed a ~ 13% increase in *V*_0_ when 50 mM Pi was added to the activating solution. However, this concentration is much higher than the Pi dissociation constant (1/*K*_5_, Tables 2, 3), hence it may not be a specific effect on CBs. In any event, they have not characterized this increase in terms of elementary steps.

A further challenge with isotonic experiments includes the need for extensive modeling, with multiple states and transitions between the states, to interpret simple results such as force–velocity experiments, compared to the use of a simple model with isometric experiments only with 3–4 states (Fig. 1). This challenge is understandable because the complex models are needed to explain no load (*V*_0_) to maximum load (isometric state), and in-between conditions. An isometric experiment is a special case of isotonic experiment, in which *V*_0_ = 0. Furthermore, it appears that the Pi effect becomes progressively less as one approaches from maximum load (isometric) to no load conditions. The modeling of isotonic experiments requires many assumptions with multiple parameters. For instance, Caremani et al. (2015) used ~ 29 CB states and ~ 33 parameters to interpret their experimental results. This trend originated with Huxley (1957) who used only 2 CB states and 2 rate constants with 2 additional parameters to explain the force–velocity data.

I have often been asked why we do not carry out force–velocity experiments, which represent a more natural condition of muscle contraction? My answer is that with force–velocity experiments, CB cycles many times; hence, the velocity is limited by the slowest step of the CB cycle. This means that information on the faster steps is obliterated and could not be resolved. Another reason is that CBs are arranged in parallel in the half sarcomere, where all the elementary steps of the CB cycle take place. Because CB forces are additive in the half sarcomere (Eq. 6) (Kawai and Zhao 1993), if the experiment was performed with the isometric condition and force was measured, there is a possibility that individual elementary steps could be resolved. Decomposition of complex modulus into 3–4 exponential processes (Kawai and Brandt 1980; Kawai and Zhao 1993) and the assignment of each process to a step in the CB cycle (Kawai and Halvorson 1989, 1991) are good examples of this principle.

### 4.2. Model studies

While it is not in the scope of this review to cover contraction models, as mentioned in the Introduction, there was a request from one of the reviewers to touch on this subject.

Offer and Ranatunga (2020) have evaluated two models in their analyses and concluded that the model, in which Pi release occurs before force generation, fits better than the model in which Pi is released after force generation. The problems I have had with this paper were as follows. (a) There are 19–20 CB states, with 33 transitions between the states, amounting to a total of 33 parameters to fit. (b) A 95% confidence limit was not calculated for each fitted parameter, so we do not have a sense of how much each fitted parameter can be trusted. (c) There were no simulations of Pi effects on most isometric experiments. One of the authors has performed (or participated in) the [Pi] dependence studies of T-jump (Ranatunga 1999a) and pressure-release (Fortune et al. 1991) experiments with results and interpretations similar to Figs. 1B/C and 2 (force is generated before Pi release); hence, a simulation of these experiments and explanation on the cause of problems why earlier conclusions were wrong would have been needed. (d) The data examined were primarily those from force–velocity experiments. Both models fit similarly during shortening, implying that isotonic experiments are not sensitive to the Pi release step, as discussed earlier in Sect. 4. The difference between the two models occurred during elongation, in which the CBs are forcibly stretched backward during activation. Although the CB motors were not designed for this purpose, I suppose it may be possible to characterize their properties by placing backward force when there are no better data. The effects of Pi on the rate constants such as shown in Fig. 2 are better data, hence should be considered with a model simulation.

Smith (2014) proposed a model with 9–10 CB states with ~ 30 constants to determine. With his model, he proposed that force generation occurs in two steps: a fractional force develops after Pi release (after Step 5 in Fig. 1), and full force develops after isomerization of the ADP bound state (AM*ADP → AM.ADP, step 6). He had simulations of the effect of [Pi] on ATPase, tension and stiffness, which were satisfactory. He also had a simulation on caged Pi experiments of Dantzig et al. (1992) at 10°C, which fitted approximately. But the absence of simulation at 20°C, which I thought were better data (twice the active force, closer to body temperature), is puzzling. Further problem was that his model predicts *T*_5_ = 0, *T*_6_ is a fractional force, and *T*_0_ is full force (*T*_6_ < *T*_0_). This prediction is in contradiction with the data we have published on rabbit psoas (Kawai and Zhao 1993) and soleus slow-twitch fibers (Wang and Kawai 1997). These data were based on actual force measurements and reproduced in Fig. 7 below. I also had a problem (b) described above. Without 95% confidence limit, we could not tell the significance of each parameter fitted hence the reliability of the model. In the future simulations, [Pi] dependence of force transients (such as shown in Fig. 2) and the temperature effect, as well as their [ATP] dependence, are desirable.

A modeling CB cycle requires many assumptions and parameters to fit. For instance, Caremani et al. (2015) used ~ 29 CB states and ~ 33 parameters to interpret their force–velocity results; Smith (2014) used 9–10 states with ~ 30 parameters; Offer and Ranatunga (2020) used 19–20 states with 33 parameters; and Månsson (2021) used 13–14 states with ~ 17 parameters. While I respect the ingenuity of these authors to be able to handle a model with such complexities, it makes the results not as informative or useful, because the importance and the essence of these models are buried in the complexity of numerous computer calculations. It has often been said that anything can be explained if a sufficiently large number of states and transitions are incorporated (Månsson 2021). Mayer et al. (2010) said that even an elephant shape can be fitted with eight independent parameters. To counter this criticism, the evaluation of 95% confidence range of each fitted parameter is essential in fitting experimentally-observed parameters to a model. This is also called “sensitivity analysis” of fitted parameters and considered by Mijailovich et al. (2010, 2012, 2021).

My philosophy in interpreting experimental results with a model is to use the model that is as simple as possible and to minimize computer calculations. My aim is that the model and its predictions must be intuitively obvious. I believe that the beauty of doing science is describing a complex phenomenon in simple terms. Albert Einstein reportedly said: “Everything should be made as simple as possible, but not simpler” (1933) (https://graeme-47328.medium.com/simplicity-34dec5e201ac). Accordingly, if there is an area that a simple model does not fit the data, then I would expand the model slowly and slightly to explain the data. The simple model should be an approximation of a more complex model. If the simple model fits to the data and more complex model does not, this means the complex model is wrong. Conversely, if the simple model does not fit to the data and the more complex model does, this means the simple model is inadequate. If both models fit, I would choose the simple one. The charm of doing science is lost when numerous states and subsequent computer calculations are required, and the importance of the problem and possible mistakes are both lost in the chaos.

## 5. Crystallographic and cryo-EM studies

### 5.1. A molecular point of view

With successful elucidation of actin’s crystal structures (Kabsch et al. 1990) and myosin S-1 (Rayment et al. 1993b), combined with cryo-electron microscopic maps (Milligan et al. 1990) of actin and myosin interactions (Rayment et al. 1993a), significant progress has been made to work out the molecular steps of energy transduction mechanisms. MgATP enters myosin from the “front door” of the nucleotide binding pocket, and is bound to the active site secured by P-loop and several additional aa residues. ATP is then cleaved to result in primed myosin. The interaction of positively charged loop 2 of myosin’s L50K with actin’s negatively charged N-terminus initiates myosin binding to actin, triggers myosin’s actin binding cleft to close, opens Pi release tunnel, release Pi to the tunnel, and starts lever arm rotation. With regard to the timing of the Pi release and lever arm rotation (force generation), it has been argued that Pi release from the active site gates the rotation of the lever arm; therefore, Pi release occurs first before force generation happens (Houdusse and Sweeney 2016).

**Fig. 4:**
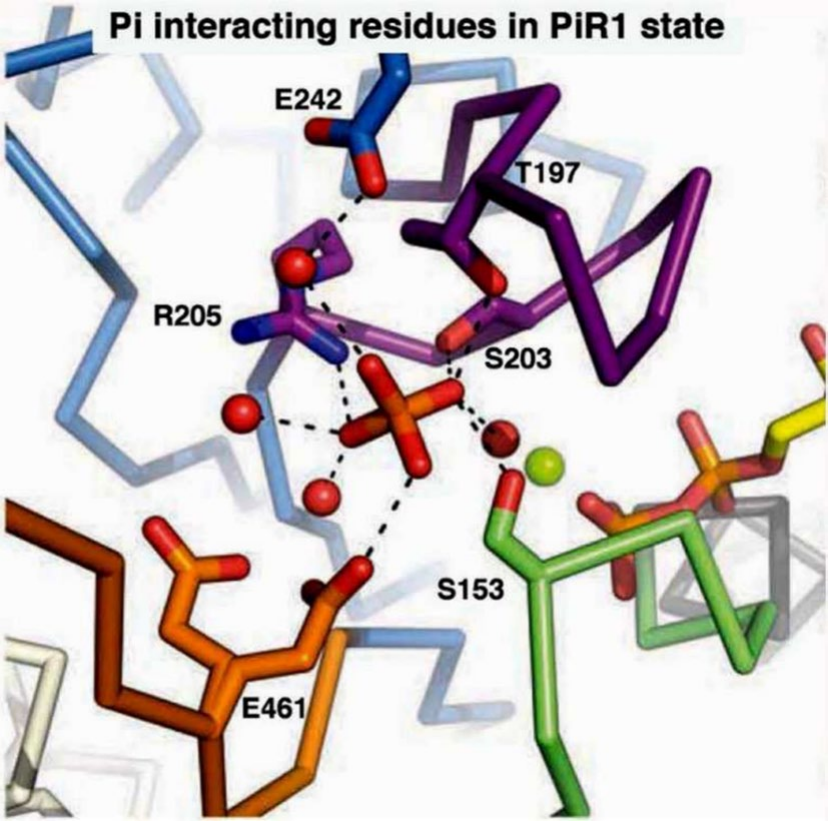
Secondary Pi binding site in Pi release tunnel. Pi at the center (4 oxygen arms in the tetrahedral arrangement are seen) is coordinated by four aa residues (S153, T197, S203 and E461) and one salt link (R205) helped by E242. aa residue numbers are based on myosin VI sequence. Red spheres are O atoms of H_2_O molecules. Reproduced from Llinas et al. (2015) with permission from *Developmental Cell* (Cell Press)

The released Pi molecule is then translocated through Pi release tunnel, and trapped by five aa residues (Llinas et al. 2015). The trapped Pi must be a stable intermediate state, because its four electron clouds of O atoms in tetrahedron configuration resonate with electron clouds of the side chains of S153, T197, S203 and E461, helped by an ionic interaction between R205 and Pi (~ 1:1 mixture of H_2_PO_4_^−^ and HPO_4_^2−^ at pH 7) to form a salt link (Fig. 4). In myosin motors, the back door is used as the Pi release channel, because the “front door” is blocked by ADP binding to the active site (Yount et al. 1995). Moretto et al. (2022) proposed that Pi release occurs first followed by the swing of the lever arm (force generation), but Pi release to the solution is delayed because of its binding to 2–3 additional Pi binding sites along the Pi escape channel in the backdoor, based on the results of atomic force microscopy (AFM). These proposals are not in conflict with experimental results shown in Fig. 2, or CB scheme in Fig. 1B/C, because physiological and biochemical experiments do not discriminate where Pi is bound to myosin.

One challenge in studying the molecular steps of the energy transduction process has been capturing the primed (prepower stroke) actomyosin state (strongly attached AM.ADP.Pi before power stroke occurs), because when this state is formed, it quickly transits to the post-power stroke state. Recently Klebl et al. (2025) observed time-resolved cryo-EM of myosin V double mutants with S217A in switch 1 and a Δ594–597 deletion (DDEK) in loop 2. S217A slows this transition (Forgacs et al. 2009), and Δ594–597 increases the affinity of primed myosin (M.ADP.Pi) to F-actin by 10 times (Yengo and Sweeney 2004). In in vitro motility assays, double mutated myosin is fully functional. When ATP is cleaved at the myosin’s active site, the Pi release tunnel in the back door is closed by a salt bridge between R219 (switch 1) and E442 (switch 2). Rapid mixing of primed myosin and F-actin followed by plunge freezing, Klebl et al. (2025) identified the prepower stroke state at 10 ms. The Cryo-EM image was examined at a 4.4 Å resolution. The primed myosin binds to the N-terminus of actin in the lower 50 kDa subdomain (L50K) with the cleft open, and this binding promotes rotation of the upper 50 kD subdomain (U50K) to close the cleft. The cleft closure displaces switch 1 and the P-loop (active site) away from switch 2 to break the salt bridge between R219 and E442, so that Pi is ready to escape. This is followed by a lever arm swing and Pi release (postpower stroke state), captured at 120 ms; the cryo-EM image was analyzed at a resolution of 4.2 Å. However, from this result we cannot determine the exact order of the power stroke and the Pi release steps. If it were possible to capture an intermediate (and stable) state between 10- and 120-ms points, this would shed a further light on this debate. To be consistent with physiological experiments, the power stroke and the Pi release steps may appear to happen simultaneously, because Pi release (Step 5) is faster than force generation (Step 4) in Fig. 1B/C.

### 5.2. Correlation with physiology/single molecule studies

To compare these molecular steps with physiological and single molecule studies, the primed and actin-bound myosin state must correspond to the AM.ADP.Pi state (X_4_, no force) in Figs. 1B/C, 5 and 6. The Pi bound state to the secondary binding site corresponds to the AM*ADP.Pi (X_5_) state with force. The rearrangements of the converter domain and the relay helix to swing the lever arm must take place while Pi is transported from the active site to the secondary site. The completion of the power stroke may trigger Pi release from the secondary site. This results in the AM*ADP (X_6_) state with force. Consequently, the results of crystallographic/cryo-EM studies and physiological/single molecule studies complement well, and actually fits to each other perfectly. The short-lived AM.ADP.Pi state (Klebl et al. 2025) is consistent to the observation by Woody et al. (2019) on single molecules, and by Kawai and Iorga (2024) on single myofibrils.

The secondary binding of Pi was identified by soaking myosin VI crystals in 25–100 mM Pi solution, and Pi was found coordinated by 5 aa residues (Fig. 4) (Llinas et al. 2015). In this mechanism, Pi is translocated from the active site to this site and trapped. In the meantime, the lever arm swings, force is generated, and then Pi is released to the solution. From the point of view of physiology and biochemistry, which approximate sequential Pi bound myosin states with one state (Fig. 1B/C), it appears as if Pi is released after force is generated, and as observed such as in Fig. 2, which is therefore consistent with the mechanism proposed by Houdusse and Sweeney (2016). Moretto et al. (2022) proposed that there are multiple Pi binding sites along the Pi release tunnel, which results in the same conclusion to explain physiological observations.

### 5.3. Why is Pi trapped in the secondary binding site?

**Fig. 5:**
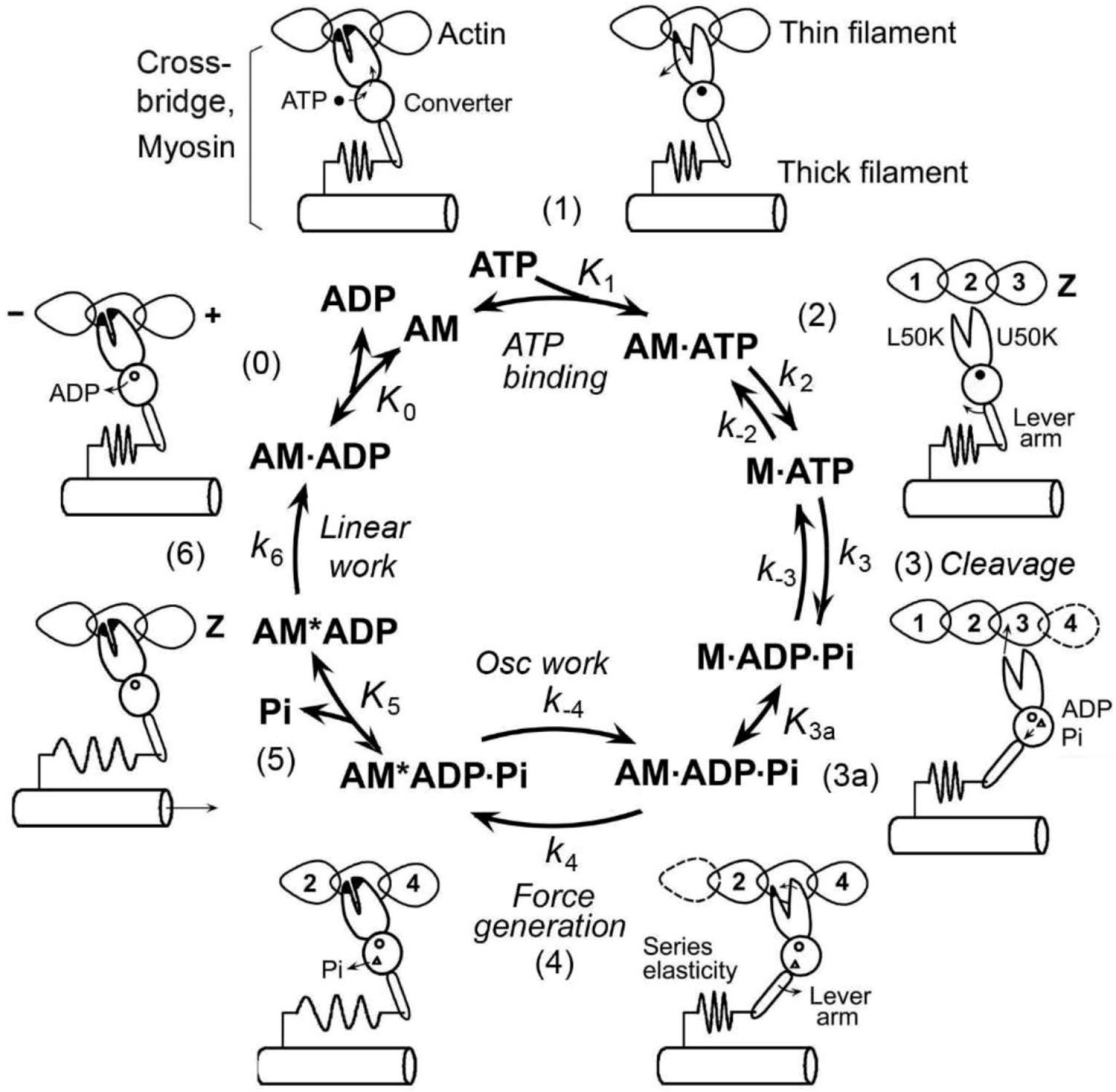
Complete cross-bridge cycle. Abbreviations are the same as those in Fig. 1. A “+” indicates the direction of the barbed end, “−" indicates the direction of the pointed end, and “Z” indicates the direction of the Z-line (barbed end). Monomeric actin is drawn to show its polarity, with individual actin molecules identified by numbers. Elementary step numbers are indicated in (), which are common to many authors. Force generation occurs at Step 4, which accompanies a closure of actin binding cleft, as predicted by Murphy et al. (1996) and as shown from crystallographic studies (Holmes et al. 2004; Málnási-Csizmadia and Kovács 2010). The dark areas (on myosin) indicate stereospecific and hydrophobic interactions between actin and myosin. The step that generates force is called the “power/working stroke” to emphasize a swing of the lever arm, which stretches series elasticity. This applies force to the external load. Actual linear work is carried out in Step 6, which transfers momentum to the load (Sect. 6.3), and the free energy stored in series elasticity is transferred to the external load as kinetic energy. Step 6 is unidirectional and is the slowest step under the isometric contraction—it rate limits the CB cycle and the ATP hydrolysis rate (Sect. 6.2). Oscillatory work is produced at Step 4 as a result of repetitive interconversion of AM*ADP⋅Pi and AM⋅ADP⋅P_i_ states. The large arrows indicate elementary steps, and small arrows indicate next movements. This CB scheme was originally published in Kawai and Candau (2010) and was relettered and republished (Kawai and Iorga 2024)

The coordination of Pi by 5 aa residues after its release from the active site may not be a coincidence. This mechanism has developed as a result of evolution, hence there has to be a reason for this. If released Pi *rebinds* to the active site, it may break the force generation process in progress and decrease the efficiency of transduction, as suggested (Scott et al. 2021). The γ-phosphate site may have to be kept empty until the swing of the lever arm is complete. Consequently, Pi must be translocated and kept away from the active site. If Pi is trapped in the release tunnel, other Pi from solution cannot access the active site. This must be, therefore, an integral part of the energy transduction mechanism. Once the lever arm swing is completed, a signal must be sent to release the bound Pi to the solution, needing a communication between the lever arm rotation and the secondary site. Pi relocation after cleavage and its release after the power stroke are depicted with cartoons in Fig. 5 (Steps 3a–4) (Kawai and Candau 2010), which is a topic of future study.

## 6. Complete CB scheme

Figure 5 shows the complete CB scheme with eight states, representing a summary of kinetic studies that were based on skinned fibers and single myofibrils. Elementary steps are indicated in (). Steps 0–2 are based on ADP and ATP studies with muscle fibers (Kawai 1978; Kawai and Halvorson 1989) and with single myofibrils (Iorga et al. 2012). Fiber and/or fibril studies depend on attached CBs, and Steps 3 and 3a occur while CBs are detached. Therefore, signals emerging from these steps are not inferred directly but are inferred from other studies, mainly from solution studies (Lymn and Taylor 1971; Bagshaw and Trentham 1973, 1974). In our physiological studies, detached states are grouped together and called the *Det* state (Kawai and Halvorson 1991; Kawai and Zhao 1993), so our physiological analysis method provides evidence on the six-state CB model. Steps 4–5 are based on Pi studies, such as those reviewed in this report. Step 6 is based on the study of the ATP hydrolysis rate (Zhao and Kawai 1994) (Sect. 6.2). Its rate can also be estimated from Process A of sinusoidal analysis results as described in Wang and Kawai (2013).

### 6.1. Sequence of events

A logical sequence of events is as follows. After the rigor complex AM is formed, MgATP moves into the nucleotide binding pocket (front door) of the myosin head and is captured by the P-loop to start the transduction process (Step 1). A part of ATP’s free energy is used to open the actin binding cleft (Conibear et al. 2003), which is opposite of cleft closure (Sect. 7), and requires energy (exothermic) to weaken the AM interaction. There may be a fast transition (Step 1b) here, which we observed as Process D with the rate constant 2π*d* in rabbit psoas fast-twitch fibers (Kawai and Zhao 1993), ferret myocardium (Kawai et al. 1993), rabbit soleus slow-twitch fibers (Wang and Kawai 1996), and mouse soleus slow-twitch fibers (Kawai et al. 2018). This is followed by CB detachment (Step 2). After the detachment, the recovery stroke (reversal of the power stroke or lever arm swing, as depicted) takes place (Steffen and Sleep 2004; Geeves and Holmes 2005). This does not require much energy because CBs do not perform work, and the structure is stabilized by switch 2 (Blanc et al. 2018). Sometime around this point, ATP cleavage takes place, producing ADP and Pi, but resulting products and high energy of hydrolysis is retained in the myosin head, called myosin product (Bagshaw and Trentham 1974) in the active site (Taylor et al. 1970). This state is depicted in Fig. 5 by M.ADP.Pi (X_3a_).

When actin thin filament is activated by Ca^2+^ and the Tn–Tpm system, the myosin head approaches actin through an electrostatic interaction between the negatively-charged (4 ×) N-terminus of actin and the positively-charged (5 ×) loop 2 of myosin’s lower 50K domain (Sutoh 1982a, 1982b; DasGupta and Reisler 1989; Sutoh et al. 1991; Furch et al. 1998; Lu et al. 2005), with the orientation of myosin possibly set by the antiparallel dipole–dipole interaction of actin E99 → R95 and D500 → K494 of myosin loop 3 (Wang et al. 2017) to transiently form the AM⋅ADP⋅P_i_ (X_4_) state (Step 3a); actin residues are numbered based on rabbit skeletal actin (Behrmann et al. 2012); myosin residues are numbered based on *Dictyostelium* myosin-IE (Durrwang et al. 2006). This is followed by a hydrophobic and stereospecific interaction between the myosin head and actin (Rayment et al. 1993a; Zhao and Kawai 1994), which accompanies a cleft closure (Murphy et al. 1996) and opening of the Pi release channel (Klebl et al. 2025). The cleft closure is followed by rearrangement of the converter and relay helix to trigger a swing of the lever arm by 60°–70° (Holmes 1998; Holmes and Geeves 2000; Geeves and Holmes 2005) to generate force, as depicted in Fig. 5 (Step 4). This step results in the force-generating AM*ADP⋅Pi state. Oscillatory work is produced by the AM⋅ADP⋅Pi and AM*ADP⋅Pi interconversion at Step 4, but this step is asymmetric and nonlinear (Abbott 1972; Kawai and Brandt 1980; Kawai et al. 2021). CBs go in the forward direction more than in the backward direction, to result in an increase in ATP hydrolysis rate, as shown by Ruegg and Tregear (1966). As soon as the lever arm swing is completed, Pi is released from the secondary binding site.

**Fig. 6:**
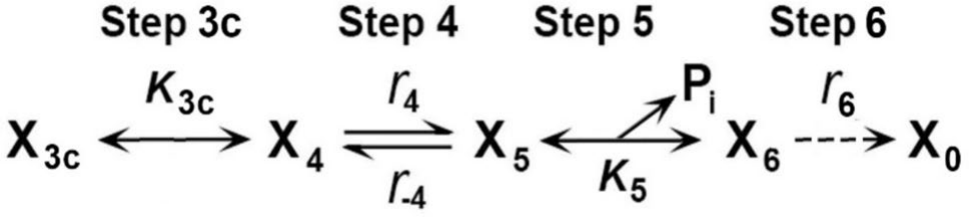
CB scheme approximation of Fig. 5 while oscillatory work (process B) is observed. X_3c_ combines states 1, 2, 3, and 3a in Fig. 5 (*X*_3c_≡*X*_1_ + *X*_2_ + *X*_3_ + *X*_3a_). Because Steps 1–3a are faster than Step 4 (Process B), they can be approximated by an equilibrium in Step 3c. *K*_3c_ is the equilibrium constant of X_3c_ ↔ X_4_ interconversion. Because Step 6 is much slower than Step 4, it can be approximated that it does not happen (*r*_6_ ≈ 0) while Process B is observed

Because the CB cycle represented in Fig. 5 has eight states and its analysis is complex, it is advantageous to simplify this cycle while oscillatory work (or Step 4) is observed. This is shown in Fig. 6, and its analysis is described in “Appendix 3”. The resulting apparent rate constant (Eq. A30) is:

17 $$2\uppi b=\frac{{K}_{3c}}{1+{K}_{3c}}{r}_{4}+\frac{{K}_{5}P}{1+{K}_{5}P}{r}_{-4}.$$

which is a slight modification of Eq. 1, to which the data fit equally well as in Fig. 2.

### 6.2. Rate-limiting step and the ATP hydrolysis rate during isometric contraction

The rate-limiting step (Step 6) is the slowest step in the CB cycle. This step can be characterized by the ATP hydrolysis rate (ATPase) measurement (Eq. 18), which is limited by the slowest step.

18 $${r}_{6}=\frac{\text{ATPase}}{{{M}_{c}X}_{6}}.$$

ATPase was measured in rabbit psoas fibers using NADH fluorescence with enzyme-coupled assays (Takashi and Putnam 1979; Griffiths et al. 1980; Kawai et al. 1987; Potma et al. 1995). *M*_c_ is the myosin head (S-1) concentration: *M*_c_ = 0.2–0.21 mM in rabbit psoas fibers, as reported by Tregear and Squire (1973) and Murakami and Uchida (1985). *X*_6_ is calculated from Eq. 4, based on equilibrium constants. These are substituted in Eq. 18 to result in *r*_6_ = 16 ± 1 s^−1^ in rabbit psoas fibers at 20°C (Zhao and Kawai 1994). At this condition, *X*_6_ = 0.34 (their Fig. 9), so *r*_6_*X*_6_ = 5.4 ± 0.3 s^−1^. *r*_6_*X*_6_ is the turn-over rate, and it compares to 13 s^−1^ (12°C) (Ferenczi and Spencer 1988), 5.9 s^−1^ at 20°C (Hilber et al. 2001), and 2.0 ± 0.2 s^−1^ at 15°C (Potma et al. 1995) in rabbit psoas fibers. In rabbit soleus (slow-twitch) fibers, it is 0.25 ± 0.02 s^−1^ at 15°C (Potma et al. 1995). In contrast, in myosin V it is 25 s^−1^ at 25°C (Gunther et al. 2020; Scott et al. 2021) with single molecule assays, and 33 ± 5 s^−1^ at 25°C (Trivedi et al. 2015) with stopped flow analysis of purified proteins.

It has been thought that Step 6 may not rate limit in shortening muscles, because the ADP release step is relatively fast in solution studies, which mimics the unloaded condition (Nyitrai and Geeves 2004; Bloemink and Geeves 2011). Also, it makes sense that as soon as the load is lifted, there is no need to keep the CB cycle paused at Step 6, and that ADP be released at this point to advance the CB cycle. Consequently, *r*_6_ must become larger (faster) during shortening (see also Sect. 4.1). This may explain the Fenn Effect that (Fenn 1924; Hill 1938) observed some time ago—the idea that more energy is utilized when an active muscle shortens. Feng (1932) reported complementary effect when active muscles were stretched together with the mechanism that series elastic elements absorbed heat on stretch and muscle cools down at the same time (Feng effect).

Step 6 may result directly in the AM state with ADP release to close the CB cycle, or it may isomerize to the AM.ADP state, as shown in Fig. 5. The AM.ADP state, formed by the addition of ADP, may be a terminal state and a competitively inhibited state of myosin (Kawai and Halvorson 1989; Iorga et al. 2012), but this is not a serious issue and does not change Eq. 18 or any other considerations. The binding of ADP or ATP to the active site is likely to be a diffusion limited fast step, so we approximated these steps by equilibria (Kawai and Halvorson 1989).

The assignment of the rate-limiting step to Step 6 during isometric contraction is based on observations that 2π*b* increases with [Pi] (Kawai 1986; Kawai and Halvorson 1991), 2π*b* and 2π*c* increase with [MgATP] (Kawai and Zhao 1993), and 2π*c* decreases with [MgADP] (Kawai and Halvorson 1989), indicating that the rate-limiting step occurs after Pi release (Step 5), but before ADP release (Step 0) or ATP binding (Step 1). Therefore, this unequivocally places the rate-limiting step at Step 6 in fibers (Kawai and Halvorson 1991; Kawai and Zhao 1993). Sleep et al. (2005) also assigned Step 6 to be the rate-limiting step after Hilber et al. (2001), and so did Bloemink and Geeves (2011).

### 6.3. Momentum transduction

Because Step 6 is the slowest step in the CB cycle during isometric contraction and occurs right after force generation, this step is convenient to generate motion to the external load, which requires momentum (***Mo***) transduction.

19 $${\varvec{M}}{\varvec{o}}\equiv m{\varvec{V}}={\int }_{0}^{\Delta t}{\varvec{f}}\left(t\right)dt\approx \overline{{\varvec{F}} }\Delta t,$$

where *m* is the mass of the object (load), ***V*** is the velocity of the movement of the object, ***f*(***t***)** is the time course of force application to the object, $\overline{{\varvec{F}} }$ is the average force applied to the object, and *Δt* is the duration of the force application. The direction of the movement is the same as that of the applied force: ***Mo***, ***V***, ***f***(*t*), and $\overline{{\varvec{F}} }$ are vectors with the same orientation. From Eq. 19 we infer that the momentum is proportional to the duration *Δt* of force application, hence a large *Δt* is advantageous for the momentum transduction for a given force. As a result, the slowest Step 6 is where most of this transduction takes place. While momentum can be transduced as soon as force is developed, Step 4 (5–10 ms) and Step 5 (~ 2 ms) are much faster than Step 6 (~ 63 ms); hence, not much momentum transduction can occur at Steps 4 or 5. It appears that the CB cycle is tuned to produce slower motion, because this is advantageous for momentum transduction, as described above (Eq. 19). For this reason, it is interesting to observe that Step 6 is slower than Step 4, and Step 4 is slower than Step 2; thus, isomerization steps gets slower each time starting from Step 1 (Kawai and Zhao 1993).

In terms of free energy, its transduction takes place in Step 4, and the free energy as a result of ATP hydrolysis is moved from the active site to the relay helix and to the converter domain in the form of potential energy. This drives the lever arm to swing to stretch series compliance. The potential energy is then moved to the series compliance, which is experienced as force generation. The free energy (*E*) stored in series elasticity is transferred to the external load as kinetic energy (*E* = 0.5 *mV*^2^), but this quantity does not provide with the direction of the movement (Kawai and Iorga 2024).

### 6.4. Force supported by each CB state

**Fig. 7:**
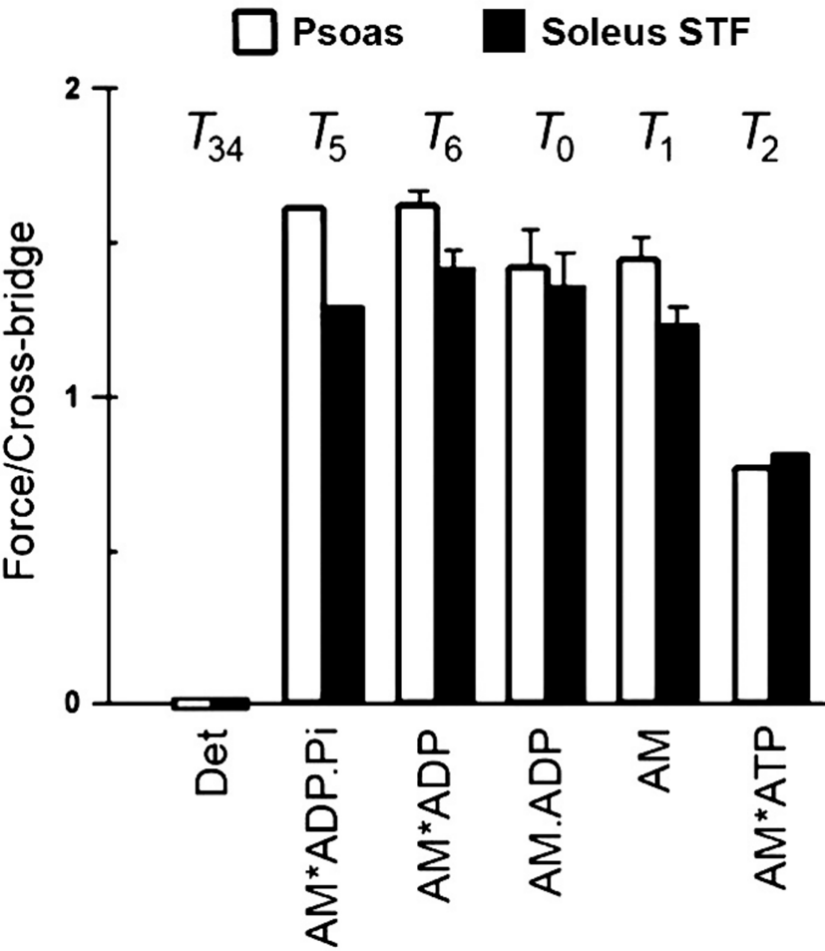
Force supported by each CB state. Rabbit psoas (fast-twitch type IID fibers) (Kawai and Zhao 1993) and rabbit soleus (slow-twitch type I fibers) (Wang and Kawai 1997). Data were taken from original publications and replotted. Data were normalized to the first control force. *Det* detached states that include M.ATP and M.ADP.Pi

The concept developed in Eqs. 1–6 can be expanded to include all the CB states shown in Fig. 5, so that force supported by each CB state can be deduced. The results are shown in Fig. 7 for psoas fast-twitch type IID fibers (Kawai and Zhao 1993) and soleus slow-twitch type I fibers (Wang and Kawai 1997) under the isometric contraction. This figure demonstrates that force (*T*_5_) develops in Step 4 (transition X_4_ → X_5_), and that the same force (*T*_6_ = *T*_5_) is maintained with Pi release in Step 5 (X_5_ → X_6_). Approximately the same force (*T*_0_ and *T*_1_) is maintained after ADP isomerization Step 6 (X_6_ → X_0_) and ADP release Step 0 (X_0_ → X_1_). Force drops to about half (*T*_2_) after ATP binding Step 1 (X_1_ → X_2_). These results are about the same for slow-twitch and fast-twitch fibers (Fig. 7). The reason why force does not decrease after Step 6, the point at which lifting of a load (work) is performed, is because the experiments were carried out under the isometric condition. As seen in Fig. 7, there is no extra force development on ADP release Step 0 (from *T*_0_ to *T*_1_). Consequently, this part of the mechanism may be different from the mechanism determined by single molecule studies on myosin V, which had an extra but small force generation with AM*ADP isomerization (Veigel et al. 2002). This step was later shown as a gate that is opened by the internal strain produced by the binding of the second head to coordinate the ATPase cycles of the two myosin V heads (Veigel et al. 2002). However, Trivedi et al. (2015) interpreted this step as the secondary force generation step by detecting the lever arm movement with CaM labelled myosin V using a stopped flow method. Doran et al. (2023) demonstrated 16° lever-arm swing (about 1/4 of full swing) with ADP release by using β cardiac myosin complexed with actin-Tpm filament with cryo-EM at 3.4 Å resolution.

### 6.5. Stretch activation, delayed tension, and oscillatory work

Stretch activation and/or delayed tension can be explained with the CB scheme in Figs. 5 and 6 and as follows. When a stretch is applied to a muscle fiber or a single myofibril, the stretch increases the potential energy stored in the series elastic elements, including in the attached CBs. According to the Le Châtelier principle, the first response to the stretch is to neutralize this energy increase, as described in Kawai and Halvorson (2007). Because the AM⋅ADP (X_0_), AM (X_1_), and AM⋅ATP (X_2_) states carry force (Fig. 7), and M⋅ATP (X_3_) does not, *K*_2_ increases and accelerates Steps 0–2, resulting in the increased detached state M⋅ATP (↑*X*_3_). Because Steps 3 and 3a are in equilibria with X_3_, the occupancy of the M⋅ADP⋅P_i_ (*X*_3a_) and AM⋅ADP⋅P_i_ (*X*_4_) states increase at the same time. In Fig. 6, the stretch increases *K*_3c_, which simultaneously decreases *X*_3c_ and increases *X*_4_. The increased *X*_4_ results in a delayed rise in tension because the X_4_ → X_5_ transition subsequently takes place with rate *r*_4_. In another words, the forward flux *r*_4_*X*_4_ has a delayed increase with the stretch, resulting in a delayed rise in tension.

What is important here is that *r*_2_ > *r*_4_ (2π*c* > 2π*b*) (Kawai and Halvorson 1991; Kawai and Zhao 1993); hence, Steps 0–3a appear to be at equilibria during the time interval in which Step 4 is observed, such as during oscillatory work production. Consequently, the increase in force lags behind the increase in length. For the sinusoidal length change, force likewise lags behind the length change at frequencies around *b* to result in a negative phase shift, which results in oscillatory work production. Thus, stretch activation, delayed tension, and oscillatory work have a common underlying molecular mechanism. In the two-state model proposed by Abbott (1972), he could not explain stretch activation, because he did not recognize that the detached and/or weakly attached population (*X*_4_) increases with a stretch. At the time, his model was not accepted because the stretch could not increase *r*_4_ (or *K*_4_), which is against the Le Châtelier principle. But it turns out that an increase in *r*_4_*X*_4_ was all that was needed for stretch activation to occur. Increased CB attachment following to a stretch (by 0.8%) and a delayed rise in force, was experimentally demonstrated by Steiger (1977) and Steiger et al. (1978) in intact rabbit papillary muscles stimulated by 70 mM K^+^ and 10 mM caffeine, and by measuring stiffness at 100 Hz.

If the muscle length is released, the exact opposite sequence of events happens, like a mirror image: the length release diminishes Step 2 (decreased *K*_2_, *K*_c_), reducing the CB occupancies at *X*_3_, *X*_3a_, and *X*_4_. The reduced *X*_4_ results in reduced force generation, resulting in a delayed decay of force. Sinusoidal length oscillation at the oscillatory work frequency *b* resonates with force generation Step 4, as the length is stretched and released repetitively. This resonance actually extends to Steps 1–3a, because they are faster than Step 4, and they appear to be in equilibria while oscillatory work is produced. But the forward flux of Step 4 (*r*_4_*X*_4_) must be larger than its backward flux (*r*_−4_*X*_5_) to cause a distorted sinusoidal response (increase in nonlinearity) when the length is released as observed (Abbott 1972; Kawai and Brandt 1980; Kawai et al. 2021). This distortion may reflect that the rate-limiting step *r*_6_ becomes faster (increase *r*_6_*X*_6_) with the length release so that more CB cycles toward the forward direction resulting in the increased ATP hydrolysis rate as observed (Ruegg and Tregear 1966).

## 7. The effect of temperature on the step that generates force

**Fig. 8:**
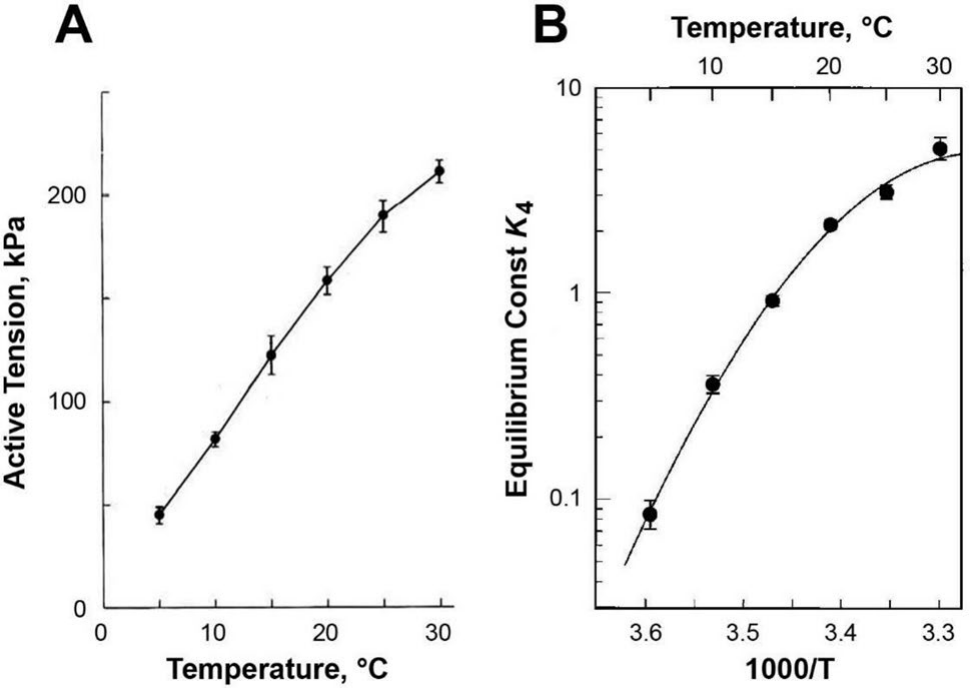
**A** Isometric tension on rabbit psoas fibers during standard activation (pCa 4.40, MgATP 5 mM, Pi 8 mM, ionic strength 200 mM, pH 7.00).** B** Equilibrium constant *K*_4_, fitted to modified Van’t Hoff equation (solid curve is based on Eq. 21 with best fit values). Replotted from Murphy et al. (1996) and Kawai (2003), original data from Zhao and Kawai (1994). Average and SEM are shown

Now let us consider why the force generation step is endothermic and absorbs heat, and why an increase in temperature causes a large increase in active force (Figs. 3, 8A). The endothermic nature of the force generation process has been reported by several authors in fast-twitch skeletal fibers (Goldman et al. 1987; Bershitsky and Tsaturyan 1992; Zhao and Kawai 1994; Ranatunga 1996), slow-twitch fibers (Ranatunga 1996; Wang and Kawai 2001), and in cardiac fibers (Ranatunga 1999b; Fujita and Kawai 2002; Lu et al. 2006). Muscle responds to the temperature increase in a way that is opposite to an automobile engine. Muscle generates a larger force when it is heated (in experiments performed at less than body temperature), whereas an automobile engine functions better when it is cooled. In the following, I will discuss why the force generation step is endothermic in muscle.

The equilibrium constant of the force generation step (*K*_4_) has a large temperature sensitivity with Q_10_ = 4.3 in rabbit psoas fibers (Table 4; Fig. 8B), which can only be explained by hydrophobic interaction resulting in a burial of large surface areas on force generation (Zhao and Kawai 1994). The most obvious place for this interaction is in the actin and myosin interface, as shown by Rayment et al. (1993a). An additional place is a large actin-binding cleft (Rayment et al. 1993b), which may close on force generation. Figure 5 depicts the cleft closure on force generation at Step 4.

### 7.1. Van’t Hoff equation and enthalpy and entropy changes

The thermodynamics of a hydrophobic interaction is discussed next. In an endothermic reaction, the equilibrium constant of the force generation step (*K*_4_) increases with temperature (Fig. 8B; Table 4) (Zhao and Kawai 1994; Wang and Kawai 2001). This results in increased force as the ambient temperature is increased in the low temperature range (Fig. 8A), peaking at around body temperature, as shown in rabbit soleus slow-twitch fibers (Wang and Kawai 2001). The endothermic nature of the force generation process was also studied by Ranatunga and his group in rabbit skeletal muscles (Ranatunga 1996; Coupland et al. 2005) with T-jump experiments. The rate constant result is fitted to the Van’t Hoff equation (Eq. 20) to deduce *ΔH*° (standard enthalpy change) and *ΔS*° (standard entropy change):

20 $$R\ln K_{4} = {-}\Delta H^{ \circ } /T + \Delta S^{ \circ } ,$$

where *R* is the gas constant, and *T* is the absolute temperature. This equation was extensively used by Ranatunga’s group, and is a general equation to fit the temperature effect. Equation 20 is a linear function of 1/*T*, so the standard linear fitting can be performed. This resulted in *ΔH*° = 103 kJ mol^−1^ and *ΔS*° = 357 J K^−1^ mol^−1^ (K ≡ Kelvin) in rabbit psoas fibers. These are large numbers that can be explained by a burial of a large hydrophobic surface area on force generation, which we initially thought to exist only in the actin and myosin interface (Zhao and Kawai 1994).

### 7.2. Further refinement of Van’t Hoff equation and the heat capacity change

In Eq. 20 one may think that *ΔH*° and *ΔS*° are independent of *T*. However, in reality they are temperature dependent (Kodama 1985). Consequently, we used the first order Taylor expansion of *ΔH*° and *ΔS*° at around *T*_r_ (an arbitrarily chosen temperature) to get more realistic results, as described in Appendix 4. The expanded Van’t Hoff equation (Eq. A34) is:

21 $${R\text{ ln}{ K}_{4}=\frac{\Delta {H}_{r}^{^\circ }}{T}+{\Delta S}_{\text{r}}^{^\circ }}+\Delta {C}_{\text{p}}\left(\frac{{T}_{r}-T}{T}-\text{ln}\frac{{T}_{r}}{T}\right),$$

22 $$\text{where } \Delta {C}_{\text{p}}\equiv {\left(\frac{\partial \Delta H^\circ }{\partial T}\right)}_{p}={\left(\frac{\partial \Delta S^\circ }{\partial lnT}\right)}_{p}.$$

*ΔC*_p_ is the heat capacity change. *ΔH*°_r_ and *ΔS*°_r_ are standard enthalpy and entropy changes, respectively, at temperature *T*_r_. Equation 21 reduces to Eq. 20 if *ΔC*_p_ = 0.

By fitting the data of *K*_4_ vs. *T* to Eq. 21 (Fig. 8B), we can obtain *ΔH*°_r_, *ΔS*°_r_, and *ΔC*_p_. This is a standard linear fitting, and three parameters with their confidence ranges are readily deduced. As this figure shows, the data fit well to Eq. 21. From this fitting we obtained *ΔH*°_r_ = 124 ± 9 kJ mol^−1^, *ΔS*°_r_ = 430 ± 30 J K^−1^ mol^−1^, and *ΔC*_p_ =  − 6.4 ± 1.8 kJ K^−1^ mol^−1^ (± 95% confidence range) in rabbit psoas fibers at *T*_r_≡288.15 K (15 °C) (Murphy et al. 1996; Kawai 2003). These results demonstrate that *ΔC*_p_ is a large negative number.

### 7.3. Burial of hydrophobic surface area

Now let us consider what happens when two molecules interact with hydrophobic aa residues. The side chains of hydrophobic residues repel H_2_O; hence, H_2_O molecules surrounding the hydrophobic residues line up around them. In a sense, the H_2_O molecules are structured and behave similarly to thin ice crystals; their mobility is limited compared to that in solution, and they form “cages” around the hydrophobic residues with low mobility (low entropy) and high heat capacity (Kodama 1985). When two hydrophobic residues interact, the structured H_2_O molecules must be melted, thereby the system absorbs the heat (*ΔH*° > 0); hence, the reaction is endothermic. The melted H_2_O molecules go into the solution phase, start full Brownian motion, and assume many more conformations, thereby entropy increases (*ΔS*° > 0). At the same time, the macromolecules lose their cage, thereby the heat capacity decreases (*ΔC*_p_ < 0). If the hydrophobic interaction is in a confined space, an efflux of H_2_O molecules follows.

It is possible to make the above analysis more quantitative and to calculate the accessible surface area (ASA) of interacting macromolecules. Equation 23 is the summary of the results (see Appendix 5, Eq. A42) needed to calculate the change of hydrophobic ASA (*ΔA*_HP_) buried by macromolecular interactions (Murphy and Gill 1991).

23 $$\Delta A_{{{\text{HP}}}} = \zeta \Delta H_{{\text{r}}}^{ \circ } + \gamma \Delta C_{{\text{p}}} ,$$

where *ζ* = 0.0401 ± 0.0052 kJ^−1^ mol nm^2^, and *γ* = 8.73 ± 0.50 kJ^−1^ K mol nm^2^ at *T*_r_ = 288.15 K (15 °C). These coefficients were determined experimentally by using model compounds (Murphy and Gill 1991), as shown in Appendix 5.

A similar relationship exists for the change of ionic ASA (*ΔA*_I_):

24 $$\Delta A_{I} = \xi \Delta H_{{\text{r}}}^{ \circ } + \varphi \Delta C_{{\text{p}}} ,$$

where *ξ* = 0.0685 ± 0.0056 kJ^−1^ mol nm^2^, and *φ* = 5.82 ± 0.48 kJ^−1^ K mol nm^2^ at *T*_r_ = 288.15 K (Appendix 5, Eq. A42) (Murphy and Gill 1991).

From Eq. 23, we calculated *ΔA*_HP_ =  − 51 ± 12 nm^2^, and from Eq. 24, *ΔA*_I_ =  − 29 ± 11 nm^2^, totaling *ΔA*_tot_≡*ΔA*_HP_ + *ΔA*_I_ =  − 80 ± 16 nm^2^ (Murphy et al. 1996) with error propagation (Bevington and Robinson 1992).

The question to ask then is where do these ASA changes come from? The most obvious place is the actin and myosin interface. Analysis by Rayment et al. (1993a) based on X-ray crystallographic studies of actin (Kabsch et al. 1990) and myosin (Rayment et al. 1993b) demonstrated that aa residues in myosin (P529, M530, I535, M541, F542, P543, T626, Q647) and actin (A144, I341, I345, L349, F352) are involved in stereospecific and hydrophobic interactions that fit to the actomyosin contour derived by cryo-EM (Milligan et al. 1990). The combined ASA of these aa residues is 16 nm^2^, only 31% of the *ΔA*_HP_ (51 nm^2^) observed from our thermodynamic analysis.

### 7.4. Where does unaccounted surface area come from?

The next question is where does the unaccounted ASA (51 nm^2^ − 16 nm^2^ = 35 nm^2^) come from? It must come from a conformational change related to force generation, and the most likely place is the myosin head, which has a large actin binding cleft (Rayment et al. 1993b). We suggested that this cleft must close on force generation to account for the unexplained burial of the large hydrophobic surface area (Murphy et al. 1996). If the hydrophobic aa residues within the cleft interact to close the cleft, the extra surface area change can be explained. The cleft closure on force generation was later confirmed by crystallographic structures using myosin V (Holmes et al. 2004; Málnási-Csizmadia and Kovács 2010); and by cryo-EM studies on myosin V (Klebl et al. 2025). This is a good story of a prediction based on careful biophysical analysis of experimental results on fibers, and confirmation of the prediction using crystallographic studies 8–14 years later. The equilibrium constant of the force generation step *K*_4_ could have contributions from the steps that occurs during CBs are detached, notably the ATP cleavage Step 3. This influences Step 3c in Fig. 6 if it is faster than Step 4, and the rate constant measured by *r*_4_ actually includes this step (Eq. 17).

As the above discussion demonstrates, the endothermic nature of the force generation step originates from intermolecular and intramolecular hydrophobic interactions. The hydrophobic interaction is effective only for a short distance (order of 1 Å); hence, for it to be significant, it must be stereospecific, and many hydrophobic aa groups must work together. The reason that temperature sensitivity increases as animals climb up in the evolutionary tree (Rall and Woledge 1990) may indicate that as animals evolved they developed more hydrophobic interactions at the force generation step and built increasingly stronger muscles, thereby winning the competition in terrestrial life.

### 7.5. Temperature change does not affect force/CB

It must be emphasized that our biophysical data are consistent with the hypothesis that temperature increase accelerates the rate constant of the force generation step (*r*_4_, Zhao and Kawai 1994; Wang and Kawai 2001) resulting an increase in its equilibrium constant (*K*_4_, Fig. 8B), because its reversal step (*r*_−4_) is not as much temperature sensitive (Table 5). This causes a shift of CB populations toward more attached, force-generating states without affecting force/CB; this conclusion does not require stiffness measurements, although our stiffness measurements are consistent to this (Zhao and Kawai 1994; Wang and Kawai 2001). At the same time, our in vitro motility assay has demonstrated that force/CB remains the same when the temperature is changed (Kawai et al. 2006). The observation that force is increased in T-jump experiments (Fig. 3A) (Bershitsky and Tsaturyan 2002) is consistent with both the prediction based on these assumptions (Fig. 3B) (Kawai 2003) and the measurement of individual kinetic constants at six different temperatures (Zhao and Kawai 1994). The underlying molecular mechanism is hydrophobic interaction between actin and myosin, and cleft closure of myosin molecule on force generation. Conversely, the hydrophobic interaction causes the endothermic reaction which increases *K*_4_ with the temperature that results in higher force. These are inseparable chain of events.

## 8. Commonalities and differences between physiological and crystal/cryo-EM studies

It is satisfying to recognize that a general agreement has been reached between physiology and biochemistry in the order of the chemical and mechanical steps involved in the CB cycle, as represented by Fig. 5. The alternation of chemical and mechanical steps was one thing pointed out by Huxley (1980). It is also satisfying to note that there is agreement on the order of the force generation and Pi release steps in physiology, in single molecule, and stopped flow studies. It is also pleasing to recognize these studies and crystallographic and cryo-EM studies complement to each other in terms of the molecular mechanisms of contraction. What it appeared as the controversy initially was actually caused by the definition of terms “Pi release step”. Physiologists and biochemists meant “from myosin”, whereas crystallographers meant “from the active site”. With the identification of the secondary binding site in myosin’s back door, a stable Pi bound state is recognized (Fig. 4) which shields the active site. The transit of Pi from the active site to the secondary site together with lever arm rotation is “isomerization” of the AM.ADP.Pi state, or called “conformational change”. Consequently, physiologists must have observed the Pi release from the secondary binding site from myosin.

There are, however, some differences between various measurements. These differences must be worth considering when correlating results from different approaches.

(a) Among different types of studies, there are vast differences in the materials used and their preparations. (b) In crystal studies, myosin is only S-1 portion without actin, tropomyosin (Tpm), or troponin (Tn), whereas some cryo-EM studies used actin and Tpm. Consequently, myosin may not have proper interactions with other macromolecules. (c) In cryo-EM and crystal studies, not enough data points can be gathered in the time sequence, so unknown portions were extrapolated. (d) Crystal and cryo-EM studies cannot measure force. (e) In physiological studies, results are based on ensemble averages of numerous molecules (10^7^–10^12^), each of which goes into a stochastic reaction, whereas in single molecule studies, the results are based on a limited number of single events (< 10^3^).

In reference to (a), among different types of studies, there are vast differences in the materials used and their preparations. For example, myosin II is used for skinned fiber/myofibril studies, myosins II and V are used for single molecules, and myosins II, V and VI are used for cryo-EM and crystal studies. It has been assumed that the mechanism of force generation through lever arm rotation is common in these three motors, and that the tail of myosin II does not affect the motor function. It has been known that actin–myosin interface is conserved among various myosins.

In reference to (b), the absence of participating macromolecules—Tpm, and Tn with Ca^2+^—is significant. So far, crystallization of myosin with actin has not been possible. In solution studies it has been known that the ATP hydrolysis rate increases > 30-fold when actin is added to myosin (Taylor and Sleep 1976; White et al. 1997); in skinned fibers ATPase increase by at least 17-fold with activation (Kawai et al. 1987), suggesting that there is a change in myosin conformation when interacting with actin. It is also known that in solution studies ATPase increases further with the addition of Tpm (Murray and Weber 1981). In skinned fiber studies, the addition of Tpm to reconstituted actin filaments increases CB force 2-fold (Fujita et al. 2002), indicating that both actin and Tpm modify myosin conformation. The inclusion of Tn with Ca^2+^ further increases isometric force (Fujita et al. 2002), indicating a further change in myosin. Together, these observations suggest a presence of consorted allosteric interactions among myosin, actin, Tpm, and Tn + Ca^2+^, or even within the myosin molecule itself. In fact, it is known from cryo-EM studies that actin binding to myosin moves its switch I (and/or switch II) away so that Pi can escape through Pi release tunnel (Geeves and Holmes 2005; Llinas et al. 2015), implying that the Pi release step is controlled by actin binding. This mechanism explains the increase in the ATPase rate as actin is bound to myosin. But the effect of actin may not be limited to the Pi release tunnel.

In reference to (c), extrapolated portions in interpreting the structural data are based on educated guesses and not observations, and not necessarily the truth. Therefore, a clear distinction must be made between theory and experimental results. In this sense, a recent report by Klebl et al. (2025) is a step forward: they have captured the primed AM.ADP.Pi state with the cleft closed and the Pi release tunnel open, but before the lever arm swing or Pi release to the solution.

In reference to (d), in crystal and cryo-EM studies, a swing of the lever arm is used in place of force, which is reasonable and justified. This swing stretches series elasticity, which is registered as a force increase. In physiological studies, force was actually measured under the presence of all participating macromolecules, which is a strength. It is most likely that swing of the lever arm and the force generation are proportionately related by the Hooke’s Law, but the elasticity may be nonlinear.

In reference to (e), in physiological studies only ensemble average of numerous (millions to trillions) molecules are used. Single molecule studies require a large number of observations approaching a thousand. But if one focuses on several hundred molecules, each one goes into a chain of stochastic reactions. Can their average represent the total population of millions to trillions? Hopefully, the answer is “yes”. This problem still remains to be solved in the future work. One worry may be that such measurements have upper and lower limits in the range of observation due to instrument’s capabilities, hence faster and/or slower rates are truncated which might give a different impression. For now, it may be proper to use “elementary steps” for physiological studies of ensemble averaged specimens, and “molecular steps” for single molecule, cryo-EM and crystal studies.

The result of the report by Klebl et al. (2025) may be interpreted to mean that the swing of the lever arm and Pi release steps take place simultaneously. This is actually consistent with the CB scheme shown in Figs. 1B/C and 6, in which Step 5 is fast, therefore, the appearance of X_5_ (AM*ADP.Pi) and X_6_ (AM*ADP) may be simultaneous. To provide evidence as to which comes out really first (force or Pi), experiments need to be designed at varying [Pi] to study how their ratio (*X*_5_:*X*_6_) changes depending on [Pi].

## 9. Conclusion

Studies using muscle fibers and single myofibrils under the isometric condition, as well as single molecule studies, have suggested unequivocally that force is generated before P_i_ release, and the same force is maintained after P_i_ release. These studies were carried out in fast-twitch fibers, slow-twitch fibers, cardiac fibers, single myofibrils, single molecules, and isolated proteins. The CB models used to arrive at this conclusion are based on 3–4 CB states and are simple and straight forward. Experiments were carried out in the presence of all contractile proteins allowing steric interactions, under the physiological ionic strength solutions. Numerous works have been published in this line of research with good statistics. An important reminder here is that the definition of Pi release or its binding is from/to myosin molecule as a whole, and it does not mean the active site of myosin in which ATP is bound and cleaved. Because there is a specific and stable secondary binding site along the Pi release tunnel, release/binding of Pi from/to this site must have been measured in physiology and biochemistry as the Pi release step. Thus, these experiments demonstrate that force generation is completed by the time Pi is released from the secondary binding site.

This conclusion is not in contradiction with the model proposed by using X-ray crystallography and cryo-EM studies (Llinas et al. 2015; Houdusse and Sweeney 2016) or AFM studies (Moretto et al. 2022), which concluded that force is generated after P_i_ release. They found a secondary and stable Pi binding site along the Pi release tunnel (Fig. 4). Here the definition of “Pi release” becomes important. Crystallographers and cryo-EM researchers meant “Pi release from the active site”, where ATP is bound and cleaved. It appears that the swing of the lever arm starts as Pi is released from the active site, and completes by the time Pi is released from the secondary binding site. The sequence of these molecular events complements quite well with the CB scheme (Figs. 1B/C, 5, 6) developed from physiological and single molecule studies. The primed prepower stroke state corresponds to AM.ADP.Pi (X_4_), the Pi bound state to the secondary binding site to AM*ADP.Pi (X_5_) which generate force, and Pi released state to AM*ADP (X_6_) which retains the force generated. Consequently, force generation occurs on X_4_ → X_5_ transition in Fig. 1B/C.

The analyses on molecular steps are based on myosin crystal and cryo-EM structures mostly in the absence of regulatory proteins. In the future work, it would be desirable to include regulatory proteins Tpm and Tn with Ca^2+^ to gain more realistic pictures, but these are not easy experiments to perform. Until then, the two lines of research may have to be treated as two separate worlds, because of there are vast differences in experimental materials, conditions, and considerations including definitions of words used in their studies.

Unloaded shortening (isotonic) and length transient experiments on fibers appear to be not very sensitive to [Pi]; hence, their Pi studies cannot provide evidence about the elementary step at which step force is generated.

## Appendix 1. Analysis of the two-state model (Fig. 1A)

### A1.1. Apparent rate constant following to a step change in the experimental condition

In the kinetic scheme of Fig. 1A, the rate of gain by X_6_ is increased by the forward rate (*r*_4_*X*_4_) and decreased by the backward rate (*r*_−4_*X*_6_):

A1 $$\frac{d{X}_{6}}{dt}{=r}_{4}{X}_{4}-{r}_{-4}{X}_{6}.$$

The effect of Pi is included in *r*_−4_. Because the total is preserved:

A2 $${X}_{4}+{X}_{6}=1.$$

From Eq. A2, *X*_4_ = 1–*X*_6_. By substituting this in Eq. A1, we obtain

A3 $$\frac{d{X}_{6}}{dt}+\varepsilon {X}_{6}={r}_{4},$$

A4 $${\text{where }}\;\varepsilon \equiv r_{4} + r_{ - 4} .$$

A5 $${\text{Replace}}\;{\text{X}}\;{\text{with}}\;{\text{Y: }}\;X_{6} \equiv Y + r_{4} /\varepsilon \equiv Y + X_{60} ,$$

$${\text{where } K}_{4}\equiv \frac{{r}_{4}}{{r}_{-4}}, \text{ and } {X}_{60}\equiv \frac{{K}_{4}}{1+{K}_{4}}.$$

A6 $$\text{Then Eq}.\text{ A}3\text{ becomes, }\frac{dY}{dt}+\varepsilon Y = 0.$$

                                By rearranging Eq. A6:   $\frac{dt}{dY}=-\frac{1}{\varepsilon Y}.$

                                

A7 $$\text{By} \, \text{integration}, t=-\frac{1}{\varepsilon }{\text{log}}_{e}Y+u.$$

                                                                              

*u* is the integration constant. After rearranging Eq. A7, we get

$$Y = \, \exp \left[ { - \varepsilon \left( {t - u} \right)} \right] = \exp \left( {\varepsilon u} \right)\exp \left( {{-}\varepsilon t} \right) \, \equiv X_{61} \exp \left( { - \varepsilon t} \right),$$

where *X*_61_ ≡ exp(*εu*) is a constant.

A8 $${\text{From}}\;{\text{Eq}}{.}\;{\text{A}}5,\;X_{6} = X_{61} \exp \left( {{-}\varepsilon t} \right) + X_{60} .$$

Consequently, *ε* in Eq. A4 is the apparent rate constant. Equation A8 is in response to a sudden change such as in the length. Similarly,

A9 $${X}_{4}={X}_{41}{\text{exp}(-\varepsilon t)+X}_{40 }{\text{, and } X}_{40}\equiv \frac{1}{{1+K}_{4}}.$$

*X*_41_ and *X*_61_ are related to the integration constant *u*, and determined by the initial condition in response to a perturbation, and proportionate to the amplitude of the perturbation, such as the step size. The apparent rate constant *ε* is common for Eqs. A8 and A9. *X*_40_ and *X*_60_ are reached when equilibrium is established at *t* → ∞. In this case, d*X*_6_/*dt* = 0 in Eq. A1; therefore,* r*_4_*X*_4_ = *r*_‒4_*X*_6_.

A10 $${\text{From } \text{this } \text{we } \text{get},   K}_{4}=\frac{{X}_{6}}{{X}_{4}}=\frac{{X}_{60}}{{X}_{40}}.$$

Equation A10 is the Mass Action Law.

### A1.2. Response to sinusoidal length changes

To solve a condition with external sinusoidal length oscillation, Eq. A3 is modified to Eq. A11 (Kawai and Halvorson 2007).

A11 $$\frac{d{X}_{6}}{dt}+\varepsilon {X}_{6}=A\text{ exp}\left(\omega ti\right),$$

where *ω* is the angular frequency (*ω* = 2p*f*; *f* = frequency) of the oscillation, *t* is time, and $i=\sqrt{-1}$*. A* is a constant proportionate to the amplitude of the length oscillation, and exp(*ωti*) is the most general form of the sinewave (Kawai 2018a). If complex nomenclature needs to be avoided, the real part of Eq. A11 can be used with Euler’s relation: exp(*ωti*) = cos(*ωt*) + *i* sin(*ωt*). The solution of Eq. A11 is expressed by the sum of two functions:

A12 $$X_{6} \left( t \right) \equiv Y\left( t \right) + Z\left( t \right),$$

where *Y*(*t*) is the general solution that satisfies Eq. A6 with an integration constant. *Z*(*t*) is any function that satisfies Eq. A11: we try the following function.

A13 $$Z\left( t \right) = B\exp \left( {\omega ti} \right).$$

By substituting Eq. A13 in Eq. A12, and further substituting it into Eq. A11, we get:

A14 $$B=\frac{A}{\varepsilon +\omega i}.$$

This means that if we set *B* as in Eq. A14, then Eq. A13 satisfies Eq. A11. Consequently, by including and considering Eqs. A6–A8, the final outcome is,

A15 $${X}_{6}(t)={X}_{61}\text{ exp}\left(-\varepsilon t\right)+\frac{A}{\varepsilon +\omega i}\text{ exp}\left(\omega ti\right).$$

A16 $$\text{Now we can rewrite,  }\frac{1}{\varepsilon +\omega i}\equiv C\text{ exp}\left(-\theta i\right),$$

A17 $$C \equiv \frac{1}{\left|\varepsilon +i\omega \right|}= \frac{1}{\sqrt{{\varepsilon }^{2}+{\omega }^{2}}}, \text{ and } \text{ tan}\theta \equiv \frac{\omega }{\varepsilon }.$$

The right side of Eq. A16 is the polar coordinate expression of the left side (Kawai 2018a) with the length *C* and angle *θ* (0° < *θ* < 90°), as defined in Eq. A17. From Eqs. A15 and A16,

A18 $$X_{6} \left( t \right) = X_{61} \exp \left( {{-}\varepsilon t} \right) \, + AC\exp \left[ {\left( {\omega t - \theta } \right)i} \right].$$

That is, the tension time course (Eq. A18) has two components: exponential time course (1st term), which is the same as step analysis result (Eq. A8), and a sinewave with phase shift *θ* (2nd term). In *real* arithmetic, cos if (*ωt*) is used in Eq. A11, this results in cos(*ωt* − *θ*) in Eq. A18. In sinusoidal analysis, data during the first 0.25 s of oscillation are not collected, so that the first term (*X*_61_ exp(− *εt*)) of Eq. A18 dissipates.

This analysis can be applied to the CB scheme in Fig. 1A by substituting *r*_−4_ → *r*_−4_*P,* and *K*_4_ → *K*_4_/*P*. Therefore, the apparent rate constant *ε* in Eq. A4 is:

A19 $$\varepsilon = r_{4} + r_{ - 4} P.$$

### A1.3. *k*_TR_ measurement

The same analysis method (Eqs. A1–A10) must have been applied by Brenner (1988) for* k*_TR_ analysis. Here the substitution needed is: $r_{4} \to f_{{{\text{app}}}} + f^{\prime}_{{{\text{app}}}} ,$ and *r*_−4_ → *g*_app_ in Eqs. A1–A4. The resulting rate constant (Eq. A4) is:

A20 $$k_{{{\text{TR}}}} = f_{{{\text{app}}}} + f^{\prime}_{{{\text{app}}}} + g_{{{\text{app}}}} .$$

From the discussion in Appendix 1, it is evident that the perturbation is applied to the equilibrium scheme (Fig. 1A), which is an approximation of the steady state. Equation A20 is sensitive to the fast rate constant, but it is not as sensitive to the slow rate constant. Consequently, *r*_6_ (Step 6) cannot be placed in *g*_app_ with isometric experiments.

## Appendix 2. Apparent rate constant of the CB scheme in Fig. 1B/C

In the kinetic scheme of Fig. 1B, X_5_ and X_6_ are in equilibrium (Step 5), hence they share a gain (*r*_4_*X*_4_) and a loss (*r*_−4_*X*_5_) (left side of Eq. A21), but the loss occurs only from *X*_5_ (right side of Eq. A21):

A21 $$\frac{d({X}_{5}+{X}_{6})}{dt}={r}_{4}{X}_{4}-{r}_{-4}{X}_{5}.$$

The Mass Action Law holds for Step 5:

A22 $${X}_{6}=\frac{{X}_{5}}{{K}_{5}P}.$$

The total is preserved:

A23 $${X}_{4}+{X}_{5}+{X}_{6}=1.$$

Because there are three unknowns (*X*_4_, *X*_5_, *X*_6_) and three independent equations (A21–A23), this system of equations can be solved, as follows:

A24 $$\text{From Eq}.\text{A}22, { X}_{5}={K}_{5}P{X}_{6},$$

A25 $$\text{From Eq}.\text{A}24, {X}_{5}+{X}_{6}=\left(1+{K}_{5}P\right){X}_{6},$$

A26 $$\text{From Eqs}.\text{A}3\text{ and A}5, { X}_{4}=1-{(X}_{5}+{X}_{6})=1-(1+{K}_{5}P){X}_{6}.$$

By substituting three quantities of Eqs. A4–A6 in Eq. A21, and by dividing both sides by

$$(1+{K}_{5}P)$$, we get

A27 $$\frac{d{X}_{6}}{dt}+\varepsilon {X}_{6}=\frac{{r}_{4}}{{1+K}_{5}P},$$

A28 $$\text{where }\varepsilon \equiv {r}_{4}+\frac{{K}_{5}P}{{1+K}_{5}P}{r}_{-4}.$$

By solving Eq. A27 (similar to Eqs. A3–A8),

A29 $$X_{6} = X_{61} \exp \left( {{-}\varepsilon t} \right) + X_{60} ,$$

$${X}_{60}\equiv \frac{1}{1+{K}_{5}P\left(1+\frac{1}{{K}_{4}}\right)}.$$

*X*_61_ is determined by the initial condition (immediately after perturbation), and *X*_60_ is the occupancy of *X*_6_ at equilibrium (*t* → ∞). Equation A29 shows that *ε* in Eq. A28 is the apparent rate constant of the CB scheme shown in Fig. 1B. *X*_5_ is derived from Eq. A24, and *X*_4_ is derived from Eq. A26. This analysis applies to a perturbation of any parameter of the CB scheme (*r*_4_, *r*_−4_, *K*_5_, *X*_4_, *X*_5_, *X*_6_) to result in the same apparent rate constant *ε* shown in Eq. A28 (Kawai and Halvorson 2007). This analysis method cannot be applied to the rate-limiting step. Instead, the method described in Sect. 3.3.5 should be used.

## Appendix 3. Simplification of the CB cycle while Step 4 (oscillatory work) is observed

An advantage of sinusoidal analysis is that this measurement is carried out one frequency (*f*) at a time. This means that a reaction that is faster than this frequency (> *f*) appears to be at equilibrium, whereas a reaction that is slower than this frequency (< *f*) appears not to happen during the time frame of the measurement. This consideration simplifies the complex problem of the CB cycle (Fig. 5) significantly. While Step 4 is being measured at an oscillatory work frequency around *b*, Steps 1–3a are faster than Step 4, so they look to be at equilibria. These fast equilibria can be grouped together to X_3c_, which is in equilibrium with X_4_ (Step 3c, Fig. 6). After force generation in Step 4 and Pi release in Step 5, Step 6 is a slow step (slower than Step 4) in isometric experiments; hence, it appears as if Step 6 does not happen while Step 4 is being measured. Consequently, one can consider that the cycle is open at Step 6 for transient analysis. As a result, the CB scheme in Fig. 5 can be approximated by a simpler scheme shown in Fig. 6.

The analytical form that relates the CB scheme in Fig. 6 to the observed rate constant 2π*b* is shown in Eq. 17, which is similar to Eq. 1. Equation 17 can be derived with a slight modification of the algorithm used in Appendix 2.

A30 $$2\pi b=\frac{{K}_{3c}}{1+{K}_{3c}} {r}_{4}+\frac{{K}_{5}P}{1+{K}_{5}P}{r}_{-4}.$$

Note that because the CB scheme in Fig. 6 is symmetrical with respect to Step 4, Eq. 17 (Eq. A30) is also symmetrical with respect to *r*_4_ and *r*_−4_. Equation A30 is a linear combination of *r*_4_ and *r*_−4_.

## Appendix 4. Expanded Van’t Hoff equation

Assuming that *ΔH*° and *ΔS*° of the Van’t Hoff equation (Eq. 20) are temperature dependent, we use first order Taylor expansion (Kawai 2018a) of *ΔH*° and *ΔS*° at around *T*_r_ (any given temperature):

A31 $${\Delta H}^{^\circ }\left(T\right)\approx {\Delta H}_{\text{r}}^{^\circ }+{\left(\frac{\partial \Delta H^\circ }{\partial T}\right)}_{p}\left(T-{T}_{\text{r}}\right)={\Delta H}_{\text{r}}^{^\circ }+\Delta {C}_{\text{p}}\left(T-{T}_{\text{r}}\right),$$

A32 $${{{\Delta S}^{^\circ }\approx \Delta S}_{\text{r}}^{^\circ }+\left(\frac{\partial \Delta S^\circ }{\partial \text{ ln}T}\right)}_{p}\left(\text{ln}T-{\text{ln}T}_{\text{r}}\right)={\Delta S}_{\text{r}}^{^\circ }+\Delta {C}_{\text{p}}\left(\text{ln}T-{\text{ln}T}_{\text{r}}\right),$$

A33 $$\text{where  } \Delta {C}_{\text{p}}\equiv {\left(\frac{\partial \Delta H^\circ }{\partial T}\right)}_{p}{=\left(\frac{\partial \Delta S^\circ }{\partial \text{ ln}T}\right)}_{p}.$$

*ΔC*_p_ is the heat capacity change under constant pressure (*p* = 1 atm), and *ΔH*°_r_ and *ΔS*°_r_ are standard enthalpy and entropy changes, respectively, at temperature *T*_r_. By using Eqs. A31 and A32, Eq. 20 becomes:

A34 $${R \text{ ln}{K}_{4}=-\frac{\Delta {H}_{r}^{^\circ }}{T}+\Delta S}_{\text{r}}^{^\circ }+\Delta {C}_{\text{p}}\left(\frac{{T}_{r}-T}{T}-\text{ln}\frac{{T}_{r}}{T}\right).$$

This is the expanded Van’t Hoff equation.

## Appendix 5. Surface area calculation based on ***ΔC***_p_ and ***ΔH***°

There is a relationship between ΔH* at T*≡373.15 K (100°C) and *ΔA*_I_ (accessible ionic surface area change):

A35 $$\Delta H^{*} \equiv \Delta H_{{\text{r}}}^{ \circ } + \Delta C_{{\text{p}}} \left( {T^{*} {-}T_{{\text{r}}} } \right) = \psi \Delta A_{{\text{I}}} ,$$

where *ψ* = 14.6 ± 1.2 kJ mol^−1^ nm^−2^ was experimentally determined (Murphy and Gill 1991). Equation A31 is used to derive Eq. A35. From Eq. A35,

A36 $$\Delta {A}_{\text{I}}=\frac{\Delta {H}_{r}^{^\circ } + \Delta {C}_{p} ({T}^{*}- {T}_{r})}{\psi }\equiv \xi {\Delta H}_{\text{r}}^{^\circ }+\varphi \Delta {C}_{\text{p}},$$

where *ξ*≡1/*ψ* = 0.0685 ± 0.0056 kJ^−1^ mol nm^2^, and *φ*≡(T* − *T*_r_)/*ψ* = 5.82 ± 0.48 kJ^−1^ K mol nm^2^. Therefore,

*ΔA*_I_ = $0.0685\times 124-5.82\times 6.4$ nm^2^ =  − 28.8 ± 11.0 nm^2^.

In addition, *ΔC*_p_ is related to *ΔA*_HP_ and *ΔA*_I_ by a linear combination (Murphy and Freire 1992; Murphy 1995):

A37 $$\Delta C_{{\text{p}}} = \alpha \Delta A_{{{\text{HP}}}} {-}\beta \Delta A_{{\text{I}}} ,$$

where *α* = 0.188 ± 0.008 kJ K^−1^ mol^−1^ nm^−2^ and *β* = 0.110 ± 0.010 kJ K^−1^ mol^−1^ nm^−2^ were experimentally determined (Murphy and Gill 1991). By substituting Eq. A36 into Eq. A37 and solving for *ΔA*_HP_, we get

A38 $$\Delta A_{{{\text{HP}}}} = \zeta \Delta H_{{\text{r}}}^{ \circ } + \gamma \Delta C_{{\text{p}}} ,$$

A39 $$\text{where } \zeta \equiv \frac{\beta }{\alpha \psi }= 0.0401\pm 0.0052 {\text{kJ}}^{-1}\text{ mol }{\text{nm}}^{2},$$

A40 $$\gamma \equiv \frac{1+\beta \varphi }{\alpha }=\frac{1+({T}^{*}-{T}_{r})\beta /\psi }{\alpha }= 8.73\pm 0.56 {\text{kJ}}^{-1}\text{ K mol }{\text{nm}}^{2}.$$

A41 $$\begin{aligned} {\text{Consequently}},\;\Delta A_{{{\text{HP}}}} & = \zeta \Delta H_{{\text{r}}}^{ \circ } + \gamma \Delta C_{{\text{p}}} \\ & = \, 0.0401 \times 124\;{\text{nm}}^{2} - 8.73 \times 6.4\;{\text{nm}}^{2} \\ & = \, 4.97\;{\text{nm}}^{2} {-}55.9\;{\text{nm}}^{2} = {-}50.9 \pm 12.0\;{\text{nm}}^{2} . \\ \end{aligned}$$

In summary,

A42 $$\left(\begin{array}{c}{\Delta A}_{\text{HP}}\\ {\Delta A}_{\mathbf{I}}\end{array}\right)=\left(\begin{array}{cc}\zeta & \gamma \\ \xi & \varphi \end{array}\right)\left(\begin{array}{c}{\Delta H^\circ }_{\text{r}}\\ {\Delta C}_{\text{p}}\end{array}\right).$$

Thus, the two vertical vectors are proportionally related.

From Eqs. A37 and A38,

$$\zeta_{{}} \Delta H_{{\text{r}}}^{ \circ } = - \beta \varphi \Delta A_{{{\text{HP}}}} + \beta \gamma \Delta A_{{\text{I}}}$$

$$\therefore \Delta {H}_{r}^{o}=\alpha \psi ( - \varphi {A}_{\text{HP}}+ \gamma {A}_{\text{I}})$$

 Or $$\left(\begin{array}{c}{\Delta H^\circ }_{\text{r}}\\ {\Delta C}_{\text{p}}\end{array}\right)=\left(\begin{array}{cc}-\alpha \varphi \psi & \alpha \psi \gamma \\ \alpha & -\beta \end{array}\right)\left(\begin{array}{c}{\Delta A}_{\text{HP}}\\ {\Delta A}_{\mathbf{I}}\end{array}\right).$$ A43

From Eqs. A42 and A43, $$\left(\begin{array}{cc}-\alpha \varphi \psi & \alpha \psi \gamma \\ \alpha & -\beta \end{array}\right)\left(\begin{array}{cc}\zeta & \gamma \\ \xi & \varphi \end{array}\right)=\left(\begin{array}{cc}1& 0\\ 0& 1\end{array}\right)$$ as expected.

## Data Availability

No datasets were generated or analysed during the current study.

## Abbreviations

≡: Definition
aa: Amino acid
AM: Actomyosin
AMM: Adductor magnus muscle
ASA: Accessible surface area
ATPase: ATP hydrolysis rate
CB: Cross-bridge
*ΔA*_HP_: Accessible hydrophobic surface area change
*ΔA*_I_: Accessible ionic surface area change
*ΔC*_p_: Heat capacity change
*ΔH*°: Standard enthalpy change
*ΔS*°: Standard entropy change
*ε*: Apparent (= measured) rate constant of CB scheme in Figs. 1 and 6, *ε* = 2π*b*
EDL: Extensor digitorum longus muscle
EM: Electron microscopy
*F*(*t*): Force time course
K: Kelvin: unit of absolute temperature
*K*_4_: Equilibrium constant of force generation step
*K*_5_: Association constant of myosin and phosphate (Pi). 1/*K*_5_ is its dissociation constant
*N*_DF_: Degree of freedom
*P*: Phosphate concentration, [Pi]
Pi: Phosphate
*Q*_10_: Increase of a parameter when temperature is increased by 10 K (10°C)
*Q*_8_: Increase of a parameter when temperature is increased by 8 K (8°C). *Q*_8_≡*Q*_10_^0.8^
*Q*_5_: Increase of a parameter when temperature is increased by 5 K (5°C). *Q*_5_≡*Q*_10_^0.5^
*R*: Gas constant, *R* = 8.3144 J mol^−1^ K^−1^
*r*_4_: Rate constant of force generation Step 4
*r*_−4_: Rate constant of reversal of Step 4
*r*_6_: Rate constant of rate-limiting Step 6
SEM: Standard error of the mean
S/N: Signal-to-noise ratio
*t*: Time in second (s)
Tpm: Tropomyosin
Tn: Troponin
*T*: Absolute temperature. Unit is K (Kelvin)
*T*_j_: Force supported by the CB state X_j_. *T*_3_ = *T*_3a_ = *T*_4_ = 0 (Fig. 7)
X_j_: CB state (*j* = 0, 1, 2, 3, 3a, 4, 5, 6)
*X*_j_: Probability (occupancy) of CBs in the X_j_ state
